# Development of a multi-mycotoxin LC-MS/MS method for the determination of biomarkers in pig urine

**DOI:** 10.1007/s12550-021-00428-w

**Published:** 2021-03-26

**Authors:** Agnieszka Tkaczyk, Piotr Jedziniak

**Affiliations:** Department of Pharmacology and Toxicology, National Veterinary Research Institute, Partyzantow 57, 24-100, Pulawy, Poland

**Keywords:** Mycotoxin biomonitoring, LC-MS/MS method, Biomarkers of exposure, Bioanalytical method optimization, Enzymatic hydrolysis

## Abstract

An LC-MS/MS method has been developed for the sensitive and selective determination of 35 mycotoxins (biomarkers of exposure) in pig urine samples. Sample preparation includes creatinine adjustment (with the developed LC-UV method) with enzymatic hydrolysis of pig urine samples followed by liquid-liquid (LLE) extraction. The LLE protocol, as well as enzymatic hydrolysis for indirect mycotoxin glucuronides determination, was optimized in this study. Additionally, two other sample preparation protocols were compared with the developed LLE method: immunoaffinity columns and solid-phase extraction cartridges (Oasis HLB). The detection and quantification of the biomarkers were performed using triple quadrupole mass spectrometry.

The method was validated with regard to the guidelines specified by the EMEA (European Medicines Agency). The extraction recoveries were higher than 60% for 77% of the analytes studied, with the intra- and inter-day relative standard deviation being lower than 20% for most of the compounds at four different concentration levels. The limits of quantification ranged from 0.1 ng/mL for zearalenone and sterigmatocystin to 8 ng/mL for nivalenol. To the best knowledge of the authors, the matrix effect was evaluated for the first time in this study for six different urine samples, and the coefficient of variation was found to be lower than 15% for most analytes studied. Finally, the developed method was applied to analyse 56 pig urine samples. Deoxynivalenol (1–20 ng/mL), zearalenone (0.1–1.5 ng/mL) and ochratoxin A (1.5–15 ng/mL) were the main analytes detected in these samples. Moreover, the co-occurrence of alternariol monomethyl ether and alternariol in pig urine is reported herein for the first time.

**Supplementary information:**

The online version contains supplementary material available at 10.1007/s12550-021-00428-w.

## Introduction

Animal feed is commonly contaminated with several mycotoxins. The main sources of toxins are cereals; maize in particular is frequently contaminated with *Fusarium* toxins like deoxynivalenol (DON) and zearalenone (ZEN) (Pereira et al. 2019). However, more than 400 mycotoxins have been identified and reported to date, and regulatory limits or the maximum tolerated level guidelines in food and feed were only established for a few in recent years (Pinotti et al. 2016).

With the exception of “free” mycotoxins, modified mycotoxins are frequently detected in feed (Guerre 2016). Many of them are hydrolysed into parent compounds or released from the feed during digestion, potentially leading to adverse health effects (EFSA 2014).

Mycotoxins have many adverse effects on both humans and animals and are a significant food safety issue (Wild and Gong 2010; Schatzmayr and Streit 2013). The ingestion of mycotoxins can cause different biological reactions ranging from acute, overt diseases with a high morbidity and mortality to chronic, insidious disorders with reduced animal productivity (Pierron et al. 2016). However, acute toxicity is rather rare; most of the animals affected are exposed to low but constant levels of mycotoxins (chronic exposure).

Mycotoxin contamination levels in animal feedstuffs are not usually high enough to cause an overt disease (Oswald et al. 2005). The major problem associated with mycotoxin-contaminated animal feed is financial loss caused by changes in animal growth and immunosuppression (Bryden 2012).

Among the various farm animals, female pigs are particularly susceptible to mycotoxins, e.g. deoxynivalenol (Pestka and Smolinski 2005) and zearalenone (EFSA 2017). The exposure of pigs to mycotoxins is usually assessed through feed analysis, which has some disadvantages. It does not assess individual exposure or the possible conversion of modified mycotoxins into parent compounds, both of which contribute to overall toxicity. Therefore, monitoring the exposure of animals to mycotoxins may include not only an analysis of toxin contamination in the diet but also the analysis of toxins in biological matrices with the use of biomarkers of exposure (the parent compounds themselves and their metabolites or reaction products, the levels of which can be measured either in the body or after extraction (Vidal et al. 2018)), as a helpful and additional tool used to assess the exposure of the pig to mycotoxins.

Biomarkers are found in biological matrices at very low levels (ng/mL). Therefore, liquid chromatography coupled with tandem mass spectrometry (LC-MS/MS) has been widely used as a method for multi-mycotoxin (biomarker) detection in urine, with a high degree of selectivity and sensitivity (Solfrizzo et al. 2011, 2014; Warth et al. 2014; Mitropoulou et al. 2018).

Sample preparation in recently published methods for the determination of mycotoxin biomarkers in pig urine samples, which are based on LLE (liquid-liquid extraction) (Song et al. 2013; Guo and Ou 2015; Lauwers et al. 2019) in contrast to those based on SPE (solid-phase extraction) (Jodlbauer et al. 2000; Brezina et al. 2014), IAC (immunoaffinity columns) (Gambacorta et al. 2013) and D&S (dilute and shoot approach) (Nagl et al. 2014; Thanner et al. 2016; Binder et al. 2017) allowed for the development of sensitive (LLOQ ≤ 1 ng/mL for the majority of analytes) and multi-mycotoxin (more than 20 analytes) methods (Table S1a).

From an analytical perspective, urine is known to be a challenging matrix due to the vast differences in composition and concentrations between individual samples. Creatinine concentration in urine may be used to determine the dilution of the urine (Arndt 2009). Additionally, DON and ZEN are excreted in pig biological matrices in the free form and in conjugated forms as glucuronides, which cannot be quantified directly due to the lack of standard materials. Therefore, in most studies, urine samples were digested with β-glucuronidase to break down the conjugated forms and obtain more accurate exposure results.

The abovementioned steps (enzymatic hydrolysis and creatinine adjustment) are missing from the developed LLE methods (Song et al. 2013; Guo and Ou 2015; Lauwers et al. 2019). Additionally, the matrix effect was only reported very rarely.

Therefore, a novel optimized LLE method, including these two important steps and a wide range of analytes (35), was developed and validated in this study. To the best of the authors’ knowledge, nivalenol, citrinin, dihydrocitrinone, fusarenon-X, altertoxin I, tentoxin and hydrolysed fumonisin B_1_ were quantified in pig urine samples for the first time. The method was also compared with other procedures commonly used for mycotoxin determination in urine samples—solid-phase extraction (SPE) and immunoaffinity columns (IAC).

Finally, the validated method was applied to the analysis of biomarkers of mycotoxins in 56 pig spot urine samples.

## Material and methods

### Chemicals and reagents

Standard solutions of deoxynivalenol (DON, molecular mass: 296.32 g/mol), 3-acetyldeoxynivalenol (3-AcDON, molecular mass 341.37 g/mol), 15-acetyldeoxynivalenol (15-AcDON, molecular mass 338.35 g/mol), citrinin (CIT, molecular mass: 250.25 g/mol), diacetoxyscirpenol (DAS, molecular mass 366.41 g/mol), fusarenon-X (FUS-X, molecular mass 354.35 g/mol), zearalenone (ZEN, molecular mass 318.36 g/mol), T-2 toxin (T-2, molecular mass 466.52 g/mol), HT-2 toxin (HT-2, molecular mass 424.48 g/mol), nivalenol (NIV, molecular mass 312.32 g/mol), tentoxin (TEN, molecular mass 414.5 g/mol), alternariol (AOH, molecular mass 258.2 g/mol), alternariol monomethyl ether (AME, molecular mass 272.3 g/mol) (100 μg/mL), deepoxy-deoxynivalenol (DOM-1, molecular mass 280.32 g/mol), sterigmatocystin (STC, molecular mass 324.28 g/mol), T-2 triol (molecular mass 382.45 g/mol), ochratoxin A (OTA, molecular mass 403.81 g/mol), ochratoxin alpha (OTα, molecular mass 256.64 g/mol), altertoxin I (ATX-I, molecular mass 352.3 g/mol) (10 μg/mL) prepared in acetonitrile (ACN) and a standard solution of hydrolysed fumonisin B_1_ (HFB_1_, molecular mass 405.6 g/mol) (25 μg/mL) prepared in ACN:H_2_O (50:50, v/v) were purchased from Romer Labs Diagnostic (Tulln, Austria) (Biopure™ certified reference materials, purity (HPLC) > 98.9%) and stored at 2–8 °C (except DOM-1—which was stored at ≤ − 15 °C). Aflatoxin B_1_ (AFB_1_; molecular mass 312.27 g/mol; purity ≥ 98.0%, HPLC), aflatoxin B_2_ (AFB_2_; molecular mass: 314.29 g/mol; purity: ≥ 98.0%, HPLC), aflatoxin G_1_ (AFG_1_; molecular mass 328.27 g/mol; purity 98%, HPLC), aflatoxin M_1_ (AFM_1_; molecular mass 328.27 g/mol; purity 98%, HPLC), alpha-zearalenol (α-ZEL; molecular mass 320.38 g/mol; purity ≥ 98%, HPLC), beta-zearalenol (β-ZEL; molecular mass 320.38 g/mol; purity ≥ 98%, HPLC), alpha-zearalanol (α-ZAL; molecular mass 322.40 g/mol; purity 98%, HPLC), beta-zearalanol (β-ZAL; molecular mass 322.40 g/mol; purity 98%, HPLC), zearalanone (ZAN; molecular mass 320.38 g/mol; purity ≥ 98.0%, TLC), enniatins (ENNs): enniatin A (ENA; molecular mass 681.90 g/mol; purity: ≥ 95%, HPLC), enniatin B (ENB; molecular mass 639.82 g/mol; purity ≥ 95%, HPLC), enniatin A_1_ (ENA_1_; molecular mass 667.87 g/mol; purity ≥ 95%, HPLC) and enniatin B_1_ (ENB_1_; molecular mass 653.85 g/mol; purity ≥ 95%, HPLC) and beauvericin (BEA; molecular mass 783.95 g/mol; purity ≥ 97%, HPLC) were purchased in powder form (5 mg) from Sigma-Aldrich (Diegem, Belgium) and stored at ≤ − 15 °C. Dihydrocitrinone (DH-CIT; molecular mass 266.25 g/mol; purity 98.9%, HPLC) was purchased in powder form (1 mg) from AnalytiCon Discovery GmbH (Potsdam, Germany) and stored at ≤ − 16 °C.

Internal standards: U-[^13^C_17_]-AFLB_1_ (0.5 μg/mL), U-[^13^C_15_]-DON (25 μg/mL), U-[^13^C_20_]-OTA (10 μg/mL), U-[^13^C_24_]-T-2 (25 μg/mL), and U-[^13^C_18_]-ZEN (25 μg/mL) were purchased from Romer Labs Diagnostic (Tulln, Austria) and stored at ≤ −16 °C.

The reference materials were purchased from the European Union Reference Laboratory (EURL) for mycotoxins in food and feed (Wageningen Food Safety Research): quality control samples for zeranol and metabolites in bovine urine (2009M1761, 2009M1764).

Beta-glucuronidases from: *Helix pomatia* (type HP-2, aqueous solution, ≥ 85,000 units/mL), abalone (aqueous solution, β-glucuronidase ≥ 100,000 units/mL) and *Escherichia coli* (*E. coli*, lyophilized powder, 1,000,000–5,000,000 units/g protein)) (Type IX-A) were obtained from Sigma-Aldrich (Darmstadt, Germany) and stored at 2–8 °C (except *E. coli*—stored at −20 °C).

Acetonitrile (ACN, analytical grade and LC-MS grade), methanol (MeOH, LC-MS grade), ethyl acetate (EtAc, HPLC grade), acetic acid were purchased from J.T. Baker (Avantor Performance Materials, Deventer, Netherlands). Formic acid and ammonium acetate (LC-MS grade) were supplied by Sigma-Aldrich (Darmstadt, Germany). Magnesium sulphate (MgSO_4_) was obtained from Chempur (Piekary Slaskie, Poland). Sodium chloride (NaCl), sodium acetate (CH_3_COONa), ammonium acetate (CH_3_COONH_4_), potassium chloride (KCl), disodium phosphate (Na_2_HPO_4_), hydrochloric acid (HCl) and trifluoroacetic acid (TFA), analytical grade were from Sigma-Aldrich (Darmstadt, Germany).

Ultrapure water was produced using the Milli-Q system (Millipore, Bedford, MA, USA).

Immunoaffinity columns DZT MS-PREP® and AOF MS-PREP® were obtained from R-Biopharm Rhone Ltd. (Glasgow, UK). The columns were connected in tandem using Supelco SPE Tube Adapter 57020-U (Sigma-Aldrich, Darmstadt, Germany). Oasis HLB cartridges, 60 mg, 3 mL, were purchased from Waters (Milford, MA, USA).

Phosphate-buffered saline (PBS) was prepared every month as follows: add 8 g NaCl, 3.6 g Na_2_HPO_4_, and 0.2 g KCl, adjust the solution to the final desired pH (7.5) using HCl, and add deionized water until the volume is 1 L.

### Preparation of standard mixtures

From the individual stock standard solutions, a standard mixture was prepared in acetonitrile and stored at −20 °C. A fresh standard mixture was prepared every month at the following concentrations: DON (200 ng/mL), 3-AcDON (200 ng/mL), 15-AcDON (800 ng/mL), DOM-1 (1200 ng/mL), ZEN (10 ng/mL), α-ZEL (40 ng/mL), β-ZEL (40 ng/mL), α-ZAL (200 ng/mL), β-ZAL (300 ng/mL), ZAN (50 ng/mL), OTA (150 ng/mL), AFB_1_, AFB_2_, AFG_1_, AFM_1_ (50 ng/mL), T-2 (100 ng/mL), HT-2 (150 ng/mL), NIV (800 ng/mL), TEN (40 ng/mL), AOH (200 ng/mL), AME (20 ng/mL), ATX-I (50 ng/mL), CIT (50 ng/mL), DAS (10 ng/mL), FUS-X (400 ng/mL), STC (10 ng/mL), T-2 triol (200 ng/mL), OTα (400 ng/mL), HFB_1_ (400 ng/mL), DH-CIT (400 ng/mL) and mix ENNs + BEA (20 ng/mL).

Additionally, an internal standard mixture was prepared from the individual stock internal standard solutions and stored at −20 °C. A fresh standard mixture was prepared every month at the following concentrations: U-[^13^C_17_]-AFB_1_ (0.02 μg/mL), U-[^13^C_15_]-DON (1 μg/mL), U-[^13^C_20_]-OTA (0.4 μg/mL), U-[^13^C_24_]-T-2 (1 μg/mL) and U-[^13^C_18_]-ZEN (1 μg/mL).

### Urine samples

Urine samples (*n* = 56) were collected from pigs before they were slaughtered between January and September 2019. Every sample was from a different pig and a different herd. The samples were taken by Veterinary Inspection in Poland under the Residue Control Monitoring Program. The samples were stored in a refrigerator at < –20 °C until analysis.

### LC-MS/MS analysis

The analysis was carried out on an Agilent 1260 Infinity HPLC system (Agilent Technologies, Waldbronn, Germany) coupled to a QTRAP® 6500 mass spectrometer (AB Sciex, Foster City, CA, USA). The detection of the analytes of interest was carried out with ESI ionization in Scheduled MRM (multiple reaction monitoring) detection mode parameters set to a window width of 60 s and a target scan time of 0.4 s in negative and positive ionization mode. Analyst® software version 1.6.2 (AB Sciex, Foster City, CA, USA) was used for data acquisition and processing. Additionally, for data processing, MultiQuant™ 3.0.1 Software (AB Sciex, Foster City, CA, USA) was used.

### LC-MS/MS condition development

For each target analyte, two or three precursors to fragment ion transitions were selected. The most abundant fragment ion was used for quantification, the second/third one for confirmation purposes. The MS/MS instrumental parameters were optimized via direct infusion (flow rate of 10 µL/min) of a standard solution (10 µg/mL of each analyte, dissolved in methanol/ultrapure water (50/50; v/v) containing 5% 10 mM ammonium acetate and 0.001% acetic acid).

Next, the chromatographic conditions were developed. The initial experiments compared the performance of the following four analytical columns; Luna: C18, C18 Omega Polar, C18 Omega PS and Phenyl-Hexyl (2 × 150 mm, 3 µm, Phenomenex, Torrance, CA, USA) in combination with various methanol and acetonitrile gradients in order to achieve the highest signal intensity and the optimal shape for all mycotoxins peaks. Next, the effect of various mobile phase additives on mycotoxin signal intensities was investigated using a Luna Omega Polar column and a methanol gradient containing different concentrations of ammonium acetate (1–20 mM) acidified with acetic acid. The effect of these additives on ionization in ESI( +) and ESI( −) was determined by comparing the signal intensities and signal-to-noise ratios obtained for high concentrations of mycotoxin standard to reveal which additive provided the highest signal intensity for a given mycotoxin.

### Final developed LC-MS/MS conditions

The ESI-source parameters which were used for all measurements were as follows: source temperature 350 °C, curtain gas 35 psi, gas 1 60 psi, gas 2 35 psi. The ion spray voltage was set at + / − 4000 V, collision gas pressure (nitrogen): high. These parameters were used to perform analyses in both the positive and negative ion modes. The MRM transitions, optimum declustering potentials and collision energies selected for each transition are given in Table S1 (MRM−) and Table S2 (MRM +).

The analytical column used was a Luna Omega Polar (2 × 150 mm, 3 µm, Phenomenex, Torrance, CA, USA) column equipped with a C18 guard column (2 × 4.6 mm, ID; Phenomenex, Torrance, CA, USA) kept at 35 °C. Eluent A was 95% MeOH (5% 10 mM ammonium acetate and 0.001% acetic acid in water) and eluent B was 95% 10 mM ammonium acetate and 0.001% acetic acid in water (5% MeOH). The following gradient elution programme was run: (0–1.0 min, 100% B), (1.0–2.0 min, linear gradient to 60% A), (2.0–3.0 min, linear gradient to 80% A), (3.0–4.5 min, 80% B), (4.5–6.0 min, linear gradient to 95% A), (6.0–10 min, 95% A), (10.0–10.1 min, 95% A), (10.1–15 min, 100% B). The injection volume was 5 µL, and the flow rate was 0.45 mL/min. The autosampler temperature was set at 4 °C.

### Sample preparation development

#### Urinary creatinine determination

An HPLC-UV method, used only for urinary creatinine determination, was developed and validated (Tkaczyk and Jedziniak 2020). Dilutions to the same creatinine concentration (0.5 mg/mL) were investigated to obtain a similar matrix effect and signal/noise (S/N) values for all analytes for urine with different creatinine levels.

#### Enzymatic hydrolysis optimization

Beta-glucuronidases from different sources (Helix Pomatia (additional arylsulfatase activity), *E. coli*, and abalone) were tested on urine spiked with a mycotoxin mixture at a medium QC level (Table 1) in terms of the matrix effect and extraction recovery influence. The following pH optima were found for beta-glucuronidase from *E. coli*—6–7.5, abalone—3.8 and *Helix pomatia*—4–5.

**Table 1:** Concentration of analytes in QC samples (spiked urine) and performance characteristics of the developed method (extraction recovery (*R*_E_), apparent recovery (*R*_A_), matrix effect (SSE) for six different urine samples and the CV of the IS-normalized SSE (CV(SSE))

Analyte	LLOQ (ng/mL)	Low QC (ng/mL)	Medium QC (ng/mL)	High QC (ng/mL)	*R*_E_ (%)	*R*_A_ (%)	SSE (%)	CV (SSE) (%)
CIT	0.5	1	4	10	89.5	23.0	28.1	14.3
α-ZEL	0.4	0.8	3.2	8	110.3	70.3	69.8	13.6
β-ZEL	0.6	1.2	4.8	12	83.8	52.4	57.4	9.6
ZEN	0.1	0.2	0.8	2	75.2	66.6	86.3	0.3
β-ZAL	3	6	24	60	80.5	55.1	65.6	0.1
α-ZAL	2	4	16	40	77.1	62.3	77.1	9.9
ZAN	0.5	1	4	10	92.1	78.4	53.1	0.0
NIV	8	16	64	160	12.6	10.3	83.1	6.2
FUS-X	2	4	16	40	94.3	125.6	133.6	11.5
DOM-1	6	12	48	120	83.7	257.7	317.7	0.0
DON	2	4	16	20	66.9	197.3	294.0	3.1
AFB_1_	0.5	1	4	10	82.0	65.1	80.4	14.6
AFB_2_	0.5	1	4	10	83.5	66.7	84.2	12.8
STC	0.1	0.2	0.8	2	72.3	67.1	92.7	7.5
AFM_1_	0.5	1	4	10	77.1	76.3	96.5	12.0
AFG_1_	0.5	1	4	10	82.9	82.3	103.3	14.5
15-AcDON	8	16	64	80	97.8	207.5	334.1	15.1
3-AcDON	2	4	16	40	80.5	190.4	334.0	12.9
DAS	0.5	1	4	10	79.4	64.4	78.3	5.4
OTA	1.5	3	12	30	60.5	60.1	102.6	14.8
HT-2	1.5	3	12	30	80.1	66.8	82.3	9.8
T-2	1	2	8	20	79.2	76.3	62.0	13.8
ENB	0.2	0.4	1.6	4	52.1	61.9	115.3	22.6
ENB_1_	0.2	0.4	1.6	4	42.0	47.5	119.3	24.1
ENA_1_	0.2	0.4	1.6	4	30.8	34.2	118.7	23.8
ENA	0.2	0.4	1.6	4	31.1	26.2	134.1	22.5
BEA	0.2	0.4	1.6	4	25.1	23.9	111.0	23.3
AOH	1	2	8	20	73.7	55.3	71.7	17.6
AME	0.2	0.4	1.6	4	63.3	44.0	86.0	6.8
ALTXI	0.5	1	4	10	69.7	40.9	53.8	14.1
TEN	0.4	0.8	3.2	8	76.7	47.5	62.3	11.8
OTα	4	8	32	80	59.6	55.2	67.2	10.3
DH-CIT	4	8	32	80	79.1	215.3	89.0	14.6
T-2 triol	2	4	16	40	79.7	58.9	278.0	8.9
HFB_1_	4	8	32	80	43.1	41.9	88.9	14.5

Different volumes (20, 30, 50 µL—1700, 2550, 4250 units) of β-glucuronidase from *E. coli* were applied for the hydrolysis of urine samples (*n* = 2 in 3 replicates) contaminated with glucuronides of ZEN, DON, ZEN and α-ZEL (determined indirectly—samples were compared before and after enzymatic hydrolysis—Fig. S1) to achieve the highest signal intensity for parent compounds after enzymatic hydrolysis. An internal standard correction was also used. Then, different hydrolysis times (2, 3 and 17 h) at different temperatures (40, 50 and 60 °C) were tested.

#### LLE procedure development

The LLE procedure was optimized on urine samples (*n* = 3) spiked at a medium QC level (Table 1).

The following parameters affecting extraction efficiency were evaluated: type and volume of extraction solvent, the addition of salts and the pH of the urine. Single modifications were added to the initial extraction conditions (5 mL of EtAc—1 g NaCl, pH 7.5) in order to study every single parameter, the non-studied parameters were kept fixed.

#### Final optimized LLE procedure with ethyl acetate used for method validation

The optimum conditions obtained from the experimental design were applied: 2.5 mL of urine was transferred into a 15-mL tube, followed by the addition of 1 g NaCl and 5 mL EtAc. An extraction was performed for 30 min on a vertical shaker (200 cycles/min), followed by centrifugation for 15 min at 4500 g and −4 °C. Then, 4 mL of the ethyl acetate phase was aspirated into a new tube, 10 µL of the internal standard mixture was added and evaporated to dryness under a gentle stream of nitrogen at 45 °C. Next, 200 µL of injection solvent, which contained 50% each of eluent A and B (final developed LC–MS/MS conditions), was used to reconstitute the residue. After centrifugation for 10 min at 14,000 × g, a 200-µL volume of this filtrate was placed into vials and used for LC-MS/MS analysis. The chromatogram obtained from the pig urine sample spiked at an LLOQ level (Table 1) with mycotoxin mixture is shown in Fig. S2. The comparison of chromatograms obtained from standard solution and spiked urine is presented in Fig. S3.

In order to ensure the reliability of the results, a matrix-matched calibration curve (QC samples, Table 1), together with blank urine and a pure solvent control, was analysed for each batch of samples. The analyte concentrations were determined through the use of isotopically labelled internal standards (IS).

### Method validation

The method was validated with regard to the guidelines specified by the EMEA (2011) in terms of linearity, selectivity, sensitivity (LOD and LLOQ), accuracy, precision (intra- and inter-day variability), matrix effect and carryover.

The linearity was evaluated by drawing six-point calibration curves in the solvent and four-point calibration curves in urine (Table 1). The coefficient of determination was defined as a measure of the linearity of the calibration curves.

The selectivity of the analytical method was assessed by using six lots of blank urine from different pigs. The effect of the interfering components was considered to be acceptable if the signal intensity was less than 20% for each analyte and less than 5% for the internal standard.

Instrumental carryover was assessed by injecting blank samples after a calibration standard at the upper limit of quantification. When injected after the highest standard concentration, the cutoff values identifying a valid signal intensity, obtained for the blank sample extract and for the internal standard, were set at ≤ 20% and ≤ 5, respectively, for the LLOQ.

LOD and LLOQ were determined using matrix-matched calibration curves. LOD and LLOQ for the different components were calculated, defining a minimum S/N of 3 and 10, respectively.

In order to achieve accuracy and precision, four quality control samples (QC samples) were prepared (Table 1). Within-run and between-run accuracy and precision were determined by analysing five samples per level at four concentration levels: the LLOQ, within three times the LLOQ (low QC), around 30–50% of the calibration curve range (medium QC) and at a minimum of 75% of the upper calibration curve range (high QC), with internal-standard correction. The mean concentration should be within 15% of the nominal values for the QC samples, except for the LLOQ, which should be within 20% of the nominal value. Additionally, within-run accuracy and precision were also evaluated for two lots of urine samples.

Evaluations of the apparent recovery (*R*_A_), extraction recovery (*R*_E_) and matrix effects (signal suppression/enhancement, SSE) were performed by comparing the slopes of three calibration curves (at four concentration levels: LLOQ, low QC, medium QC and high QC): a calibration curve was prepared using mobile phase (I), matrix-matched calibration curves prepared by spiking before (II) and after (III) sample preparation, all with internal standards correction.

*R*_E_, *R*_A_ and SSE were investigated using six lots of blank urine. The IS-normalized SSE was calculated by dividing the SSE of the analyte by the SSE of the IS. The CV of the IS-normalized SSE calculated from the six lots of matrix should be lower than 15%.

For ZAN, α-ZAL, β-ZAL and β-ZEL, the trueness of the method was confirmed through the analysis of reference materials of bovine urine.

### Comparison of sample preparation protocols

Three sample preparation protocols were tested: based on liquid-liquid extraction (“LLE”), sample clean-up with multi-mycotoxins immunoaffinity columns (“IAC”), and sample clean-up with solid-phase extraction (“SPE”) cartridges (Oasis HLB).

LLE was optimized in this study (LLE procedure development). Two other protocols (IAC and SPE) were based on data provided by the producer (generic methods described by the manufacturers—IAC (R-Biopharm) and SPE (Waters)) and modified for pig urine.

Extraction recovery and matrix effect experiments were performed on three urine samples spiked at medium QC levels (Table 1) in three replicates for each sample preparation technique. Extraction recovery (*R*_E_) and SSE were calculated as described in the LLE method validation.

For every protocol, before analysis, urine samples were allowed to reach room temperature and then centrifuged for 15 min at 4500 g and −4 °C. Next, the samples were standardized by dilution to a constant creatinine level of −0.5 mg/mL (Tkaczyk and Jedziniak 2020) with PBS (pH 7.5) and incubated with β-glucuronidase from *E. coli* for 2 h.

### Sample clean-up using immunoaffinity columns

The standardized urine (2.5 mL) was diluted with 5 mL of PBS buffer, mixed and then slowly (1 mL/min) passed through the AOF-MS-PREP and DZT-MS-PREP multi-antibody IAC connected in tandem. The cartridges were then rinsed with 10 mL of water. Elution was performed with 2 × 1.5 mL methanol. Then, 10 µL of internal standard was added. The eluate was evaporated to dryness at 40 °C. The final residue was re-dissolved in 100 µL of mobile phase A and 100 µL of mobile phase B.

### Sample clean-up using Oasis HLB cartridges

The SPE protocol consisted of diluting 2.5 mL of a standardized urine sample with ultrapure water (1/2, v/v) followed by loading the diluted urine on a pre-conditioned Oasis HLB cartridge. The samples were loaded and allowed to flow through at a flow rate of one drop per second. Interfering substances were washed away with 2 mL of water. Elution was performed with 3 mL of methanol. Then, 10 µL of internal standard was added. The eluate was evaporated to dryness at 40 °C. The final residue was redissolved in 100 µL of mobile phase A and 100 µL of mobile phase B.

## Results and discussion

### Mass spectrometer parameters

During method development, it became obvious that both transitions of DON in negative ionization mode (355/265 and 355/295) had severe matrix interferences and abundant background noise in both the blank and spiked urine samples which did not allow for adequate LOD values to be obtained. Therefore, a positive ionization mode was chosen—similar to that used in other studies (Guo and Ou 2015; Wells et al. 2017; Deng et al. 2018). Moreover, the addition of ammonium acetate has proven to be necessary to support the formation of [M + NH_4_^+^] adducts, which appear to be the most predominant ions in the spectra of HT-2, T-2 triol and DAS. T-2 toxin was detected in the positive ionization mode with the sodium adduct (higher signal-to-noise values) in contrast to the majority of the published methods—with the ammonium acetate adduct (Warth et al. 2012; Guo and Ou 2015).

The ion source temperature was an important parameter to optimize for DON, ZEN, NIV, FUS-X, ENNs and BEA. A lower temperature of 250–350 °C resulted in a higher signal intensity than that found at higher temperatures. Curtain gas 40 psi enabled the achievement of the optimal signal intensity for all analytes.

### Liquid chromatography parameters

Four analytical columns, Luna: C18, C18 Omega Polar, C18 Omega PS and Phenyl-Hexyl (2 × 150 mm, 3 µm, Phenomenex), were tested. Among them, the Luna Omega Polar (2 × 150 mm, 3 µm) from Phenomenex was found to provide the best resolution and peak shape for all target compounds and was therefore selected for future studies.

Several varieties of compositions of mobile phases were tested, including organic modifiers (ACN, MeOH) and organic salts like ammonium acetate (1–20 mM) acidified with acetic acid. For most analytes, the mobile phase with MeOH resulted in a higher signal intensity, except for ZEN. Although the most suitable mobile phase for ZEN determination consisted of ACN, the highest signal intensity and optimal peak shape for DON, T-2 and HT-2 were achieved with a mobile phase of –MeOH. The baseline separation of ZEN and its metabolites was achieved when only MeOH was used as the organic mobile phase (Li et al. 2018) (Fig. S3). The use of 10 mM ammonium acetate as an additive in the mobile phase produced a higher signal for DON, T-2 and HT-2 than the use of 1, 5 and 20 mM ammonium acetate. The use of ammonium acetate resulted in a significantly higher signal intensity for the majority of mycotoxins than ammonium formate (Huybrechts et al. 2015; Deng et al. 2018).

### Optimization of sample preparation

There were two main challenges, which were solved before the optimization of the sample preparation protocol: dilution to a constant creatine level and the optimization of enzymatic hydrolysis.

#### Urinary creatinine

The first challenge was the high degree of the diversity of the results for different urine samples, because of the different water contents. Biomonitoring data are usually adjusted to a constant creatinine concentration to correct for variable dilutions among samples (O’Brien et al. 2017).

A recently developed LC-UV method was applied for creatinine determination and the samples were diluted to a constant creatinine level (0.5 mg/mL) before analysis (Tkaczyk and Jedziniak 2020). In the case of mycotoxin urinary biomarkers in pigs, there are only two studies available in which standardization for different dilutions of urine samples was used for the analysis: DON, ZEN and their metabolites (Thanner et al. 2016; Binder et al. 2017) (dilution to 0.2 mM creatinine) after the dilute and shoot approach. Once the concentration of each analyte was reported in terms of ng/mL and ng/mg creatinine, the analysis of DON, DOM-1, AFM_1_, FB_1_, ZEN, α-ZEL, β-ZEL and OTA proceeded after immunoaffinity columns were connected in tandem with solid-phase extraction columns (Gambacorta et al. 2019).

The adjustment to 0.5 mg/mL of creatinine allowed for the standardization of IS-normalized SSE for different pig urine samples—as described in detail in a recently published paper (Tkaczyk and Jedziniak 2020).

Another problem connected with the different urinary creatinine levels was the different signal-to-noise values and, therefore, different limits of detection for urine with different creatinine levels.

**Fig. 1:**
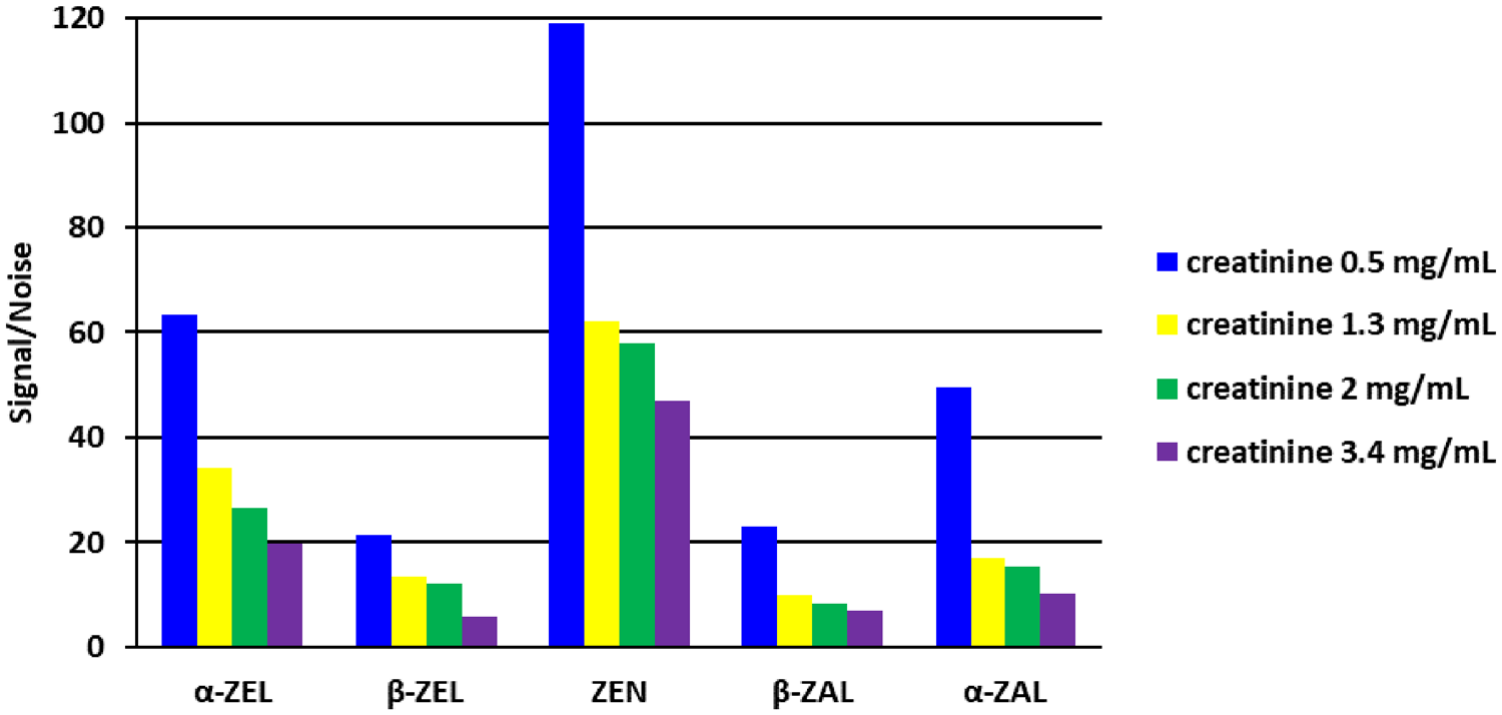
Comparison of S/N values of ZEN and its metabolites at the same spiking level for different urinary creatinine levels

The dilution to a creatinine level of 0.5 mg/mL allowed for the standardization of matrix effects and S/N values for ZEN and its metabolites, and therefore, it was possible to establish the limits of detection (LODs) (Fig. 1).

#### Enzymatic hydrolysis optimization

A comparison was made between the mycotoxin concentrations in the urine samples (*n* = 20) both before and after enzymatic hydrolysis (with beta-glucuronidase from *E. coli*, 2 h, 40 °C) which allowed for an assessment to be made of the glucuronidation rate of different mycotoxins.

Almost complete glucuronidation of ZEN was found, thereby indicating the glucuronide of ZEN as a major metabolite of ZEN in the urine of pigs. This result is consistent with other research and highlights the importance of enzymatic hydrolysis (Binder et al. 2017; Lauwers et al. 2019).

According to the best knowledge of the author, this important step has never been fully optimized before. In recent times, β-glucuronidases were only quantified for human urine samples (Liu et al. 2020). In the case of pig urine, enzymatic hydrolysis was carried out but only in three recent studies: β-glucuronidases from Helix Pomatia (Gambacorta et al. 2013, 2019; Brezina et al. 2014) and *E. coli* (Nagl et al. 2014). Similar matrix effects and extraction recoveries (RSD < 15%) were observed in the urine samples after enzymatic hydrolysis with different enzymes, with the exception of T-2 toxin. Only the use of enzyme from *E. coli* allowed for T-2 toxin determination with a satisfactory recovery (80%) compared to other sources of enzyme: *Helix pomatia* (35%) and abalone (56%). After overnight incubation with beta-glucuronidase from *Helix pomatia* and abalone, in contrast to *E. coli*, T-2 toxin could not be determined. Therefore, beta-glucuronidase from *E. coli* was chosen for hydrolysis and applied in the analysis. As shown in Fig. S4, there were low differences (RSD < 15%) in the ratios between the areas produced by the analyte and internal standard of all tested volumes of enzymes as well as the temperatures and times of enzymatic hydrolysis. Therefore, 2 h at 40 °C was chosen for enzymatic hydrolysis and applied to future analyses.

#### Sample preparation protocols

A wide variety of sample preparation methods have been reported in the literature for mycotoxins in pig urine, mainly LLE, SPE, IAC and D&S (dilute and shoot) (Table S1a). The most popular sample preparation method used for the analysis of multiple mycotoxins in pig urine samples was based on LLE. In recently published studies, LLE, ACN and EtAc or their acidified solutions with 1% FA and salts additions were used as solvents for the extraction of multiple mycotoxins from urine samples (Song et al. 2013; Sun et al. 2014; Guo and Ou 2015; Lauwers et al. 2019).

In our study, two solvents, ACN and EtAc, were tested and compared with regard to their extraction efficiency for the studied mycotoxins using urine samples (*n* = 3 in three replicates) spiked at a medium QC level (Table 1) before and after the extractions were investigated.

The best recovery ranges were obtained with EtAc (34–98%) compared to ACN (32–93%) for most of the analytes studied (except ZEN). For some mycotoxins, such as AFG_1_ and HT-2, similar recoveries were obtained with the use of both ACN and EtAc. However, the recovery of DON decreased from 98% (EtAc) to 32% when ACN was used. EtAc was also used as an extraction solvent in the recent multi-mycotoxin LC-MS method for the determination of 24 mycotoxins and their metabolites in pig urine (Lauwers et al. 2019). Another study revealed that satisfactory extraction recoveries (74.3–102.4%) were achieved for T-2, HT-2 and T-2 triol, when ACN was applied as an extraction solvent (Sun et al. 2014)—in contrast to our results (*R*_E_ < 60%). Therefore, EtAc was selected as the optimal extraction solvent.

Next, different combinations of extraction with EtAc were tested: EtAc, acidified EtAc (0.1% HCOOH), double extraction: EtAc-EtAc and EtAc-EtAc (0.1% HCOOH) and extraction with EtAc-MeOH (7.5: 5). As shown in Fig. S5, the best recovery ranges were obtained with EtAc (60–104%) for all analytes.

Unfortunately, the extraction recovery of OTA after extraction with EtAc was low (34%). After analysis of the aqueous phase, it was determined that OTA was mainly detected there (60% of spiking concentration). Therefore, the addition of different salts was tested and three of them (CH_3_COONH_4_, NaCl and MgSO_4_) resulted in a satisfactory recovery of OTA (> 80%). After the addition of MgSO_4_ and CH_3_COONH_4_, the extraction recovery of ZEN and its metabolites was much lower (45–85%) compared to NaCl (75–110%) (Fig. S6). Therefore, NaCl was selected for further experiments. In the case of pig urine, salting-out assisted LLE (SALLE) for multi-mycotoxin biomarkers (including OTA) this analysis was only applied to one study (salt—2 M MgSO_4_) (Song et al. 2013).

Then, different volumes of urine (0.5–3.5 mL) and different volumes of ethyl acetate (2.5—7.5 mL) were tested on three different urine samples spiked with mycotoxin mixture at a medium QC level (Table 1).

Finally, it was established that the optimal parameters were as follows: 2.5 mL of urine, 1 g of NaCl and 5 mL of ethyl acetate.

Then, the impact of such pH values as 3.9, 6.5 and 7.5 on the mycotoxin recoveries from the urine sample spiked at a medium QC level (Table 1) was tested. A pH value of 7.5 resulted in the highest absolute areas for ZEN and its metabolites, DON, and its metabolites, T-2, and HT-2 toxin. The highest area of OTA was achieved for the acidic pH (Fig. S7). These results are consistent with data from other biomonitoring studies in pigs (Lauwers et al. 2019). The pH value of 7.5 was applied in future analyses—before analysis, all urine samples were diluted to a constant creatinine level (0.5 mg/mL) with PBS. This pH value is also consistent with the recommended conditions for enzymatic hydrolysis with beta-glucuronidase from *E. coli*.

### Validation

The developed LLE method was successfully validated (Table 1; Table S3). The regression coefficients (*R*^2^) of the calibration curves ranged from 0.9922 to 0.9999, with deviations of less than 15% for all measured concentrations. These results indicated good linear fits for all analytes.

After analysing six lots of blank urine samples, no endogenous interferences were observed at the retention time of each analyte or internal standard. No carryover was observed.

The LOD and LLOQ values were 0.03–2 ng/mL and 0.1–8 ng/mL, respectively, with relative standard deviations (RSDs) at LOQ levels of less than 20% (*n* = 6) for all of the analytes (Table S3). The sensitivity of the method is similar compared with the previously reported multi-mycotoxin method based on LLE (Lauwers et al. 2019).

Accuracy values, expressed in terms of recoveries, ranged between 78.4 and 109.6% for intra-day accuracy and between 80.9 and 101.8% for inter-day accuracy, at all concentration levels. The intra-day and inter-day precisions (based on the RSD) were 4.4–13.1% and 6.1–20.3%, respectively (Table S3). The results met the appropriate requirements for all mycotoxins.

In addition, accuracy and precision were calculated for two different batches of urine samples (Table S3). The accuracy values ranged from between 99.5 and 100.9%, whereas the precision values were as follows: 5.3–17.9%. These values indicate the good reproducibility of the results for different urine samples.

*R*_E_, *R*_A_ and SSE were calculated for six different urine samples. The *R*_E_ values ranged from 12.6% for NIV to 119.3% for α-ZEL. The *R*_E_ value for most of the analytes (77%) was similar to those of the other LLE method (84%) which were in the range of 60–110% (Lauwers et al. 2019). The *R*_E_ values of NIV (12.6%), as well as ENNs and BEA (25.1–52.1%), were low. With the dilute-and-shoot approach, this parameter was quantified in human urine with high apparent recovery (82%) (Warth et al. 2012). Higher extraction recoveries of ENNs (73.9–80.3%) and BEA (79.9%) were achieved by other authors (Lauwers et al. 2019). In contrast to other validation guidelines (EC 2002), recovery is not addressed in the EMEA requirements. For biological matrices, it is important to demonstrate reproducibility rather than to show a higher recovery rate (FDA 2018).

SSE values of between 28.1% for CIT and 334.1% for DON were obtained. Significant signal suppression (SSE < 80%) was found for CIT, α-ZEL, β-ZEL, α-ZAL, β-ZAL, ZAN, STC, AOH, ATX-I and OTα and significant signal enhancement was found (SSE > 120%) for NIV < FUS-X, DOM-1, DON, AFG_1_, ENA, T-2 triol and HFB_1_. In contrast to other methods, it only demonstrated significant signal suppression for AOH, AME, DON, DOM-1, 3-/15-AcDON, AFB_1_ and AFM_1_ (Lauwers et al. 2019).

The results demonstrated an effective analyte extraction while showing the necessity of internal standard compensation due to a significant matrix effect for the majority of analytes and therefore a rather low apparent recovery.

For all mycotoxins, an adequate internal standard and matrix-matched calibration curves were used, resulting in validation results for accuracy and precision matching the acceptance criteria. Based on the results, internal standards with similar retention times and matrix effects were selected as reference internal standards for compounds lacking commercial internal standards (Table S1).

It is important to emphasize the CV of the IS-normalized SSE calculated from the six lots of urine samples, which was lower than 15% for most analytes (except ENNs—lower than 25%). This fact demonstrates the very good reproducibility of the method. According to the best knowledge of the author, it is the first multi-mycotoxin method for pig urine samples, in which the matrix effect was assessed in six different urine samples and the *R*_E_, *R*_A_, SSE and CV of the IS-normalized SSE (CV(SSE)) was calculated from the six lots of urine samples.

For zearalanone, α-zearalanol, β-zearalanol and β-zearalenol, the trueness of the developed method was confirmed through the analysis of reference materials of bovine urine. The experimentally determined concentrations showed a satisfactory agreement with the certified values (Table 2).

**Table 2:** Comparison between certified and measured concentrations of certified reference materials

Material	Analyte	Certified concentration (ng/mL)	Measured concentration (ng/mL) (*n* = 3)
2009M1761	β-ZEL	1.06	0.94 ± 0.11
	β-ZAL	6.63	6.56 ± 0.49
	α-ZAL	3.22	2.91 ± 0.10
	ZAN	1.09	0.95 ± 0.02
2009M1764	β-ZEL	1.59	1.35 ± 0.03
	β-ZAL	16.52	16.15 ± 0.27
	α-ZAL	8.56	8.09 ± 0.31
	ZAN	2.86	2.75 ± 0.12

#### Comparison of LLE with other sample preparation protocols (IAC and SPE)

The developed LLE method was compared with two other methods which are frequently applied in the analysis of pig urine samples. Only eight analytes were selected for this comparison because only these analytes could be determined with IAC and SPE columns.

**Fig. 2:**
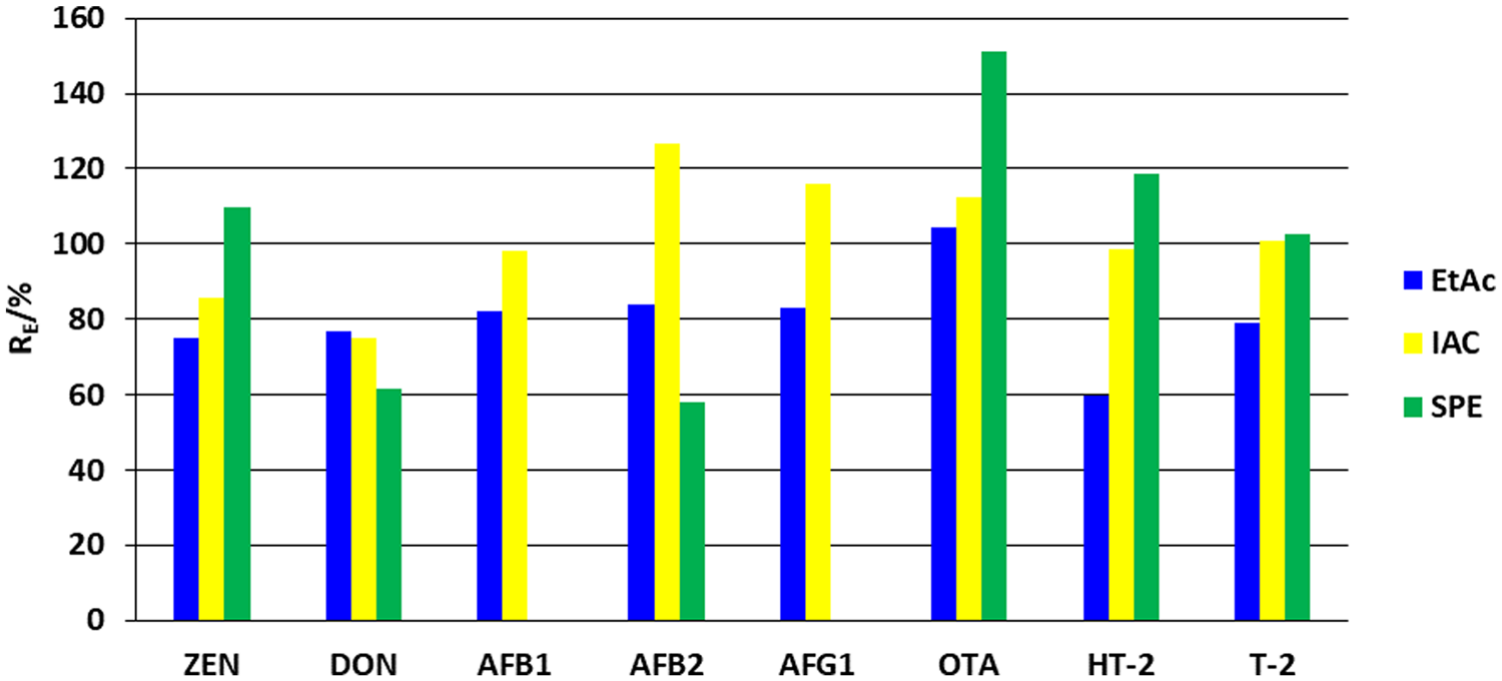
Extraction recovery performed by LLE, IAC and SPE

DON, ZEN, T-2, HT-2, AFB_1_, AFB_2_, AFG_1_ and OTA were determined with satisfactory extraction recoveries—which ranged from 75% for DON to 127% for AFB_2_ with IAC clean-up (Fig. 2). It is worth noting that CIT, NIV, STC, BEA and ENNs and metabolites such as ZAN, OTα, T-2 triol, HFB_1_ and DOM-1 could be detected using IAC columns, but with a lower sensitivity and extraction recovery (< 50%) compared to the eight analytes selected for this experiment.

In the case of LLE, the extraction recovery was in the range of 60–104% (Fig. 2). As for IAC, significant signal enhancement was observed for DON (about 300%) (Fig. S8), which was consistent for different urine samples (RSD < 20%). Although S/N values (Fig. S9) were two times lower compared to IAC, they were satisfactory to reach a sufficient LOD for 35 mycotoxins.

Sample clean-up using SPE for most compounds resulted in co-elution and interference with matrix components at this concentration range. This resulted in higher analyte LODs (2–40 ng/mL) than for LLE and IAC (Fig. S8). Some analytes like AFB_1_ and AFG_1_ could not be detected at a medium QC level (Table 1). The matrix effects for most compounds were about 50%. Due to the abovementioned reasons, this approach was not considered suitable. The IAC columns allowed for a reliable clean-up (satisfactory recoveries, high sensitivity) but for a narrow range of analytes limited to the cross-reactivity range (8 analytes) and high cost. Therefore, LLE provides an inexpensive and sufficient clean-up for a wide range of mycotoxins as an alternative to IAC, which could be applied when lower LOQs are needed.

### Analysis of pig urine samples

Once the method was validated, it was applied to investigate the occurrence of 35 analytes in 56 pig urine samples. All samples were analysed in duplicate, and the concentrations of the analytes were calculated from matrix-matched calibration curves.

As shown in Table 3, DON, ZEN, α-ZEL, β-ZEL, OTA, AOH and AME were detected in pig urine samples. The most frequently detected analyte was ZEN (75%) with a median concentration of 0.27 ng/mL—similar to other results (Jodlbauer et al. 2000; Gambacorta et al. 2019). AME had the second-highest detection rate at 73% of samples. Similar detection rates for OTA (56%) and AOH (54%) were observed. OTA and AOH were found in about half of the samples with median concentrations of 2.48 and 1.97 ng/mL, respectively.

**Table 3:** Biomarker contamination profile in 56 pig urine samples

Analyte	Positive samples > LOD (%)	Positives samples > LLOQ (%)	Mean concentration in diluted urine samples (0.5 mg/mL creatinine) (ng/mL) ± SD	Median concentration in diluted urine samples (0.5 mg/mL creatinine) (ng/mL)	Range (ng/mL)	Mean concentration (ng/mg creatinine) ± SD	Median concentration (ng/mg creatinine)	Range (ng/mg creatinine)
DON	46	26	7.02 ± 7.36	1.64	2–20	0.24 ± 0.11	0.23	0.12–0.31
ZEN	75	53	0.36 ± 0.31	0.27	0.1–1.5	0.16 ± 0.13	0.15	0.02–0.30
α-ZEL	46	4	0.54 ± 0.01	0.54	0.4–0.5	0.18 ± 0.01	0.18	0.17–0.19
β-ZEL	9	0	-	-	-	-	-	-
OTA	56	26	3.55 ± 3.41	2.48	1.5–15	1.74 ± 2.34	0.90	0.15–9.81
AOH	54	11	2.35 ± 1.54	1.97	1–4.4	0.99 ± 0.57	0.95	0.22–1.89
AME	73	16	0.37 ± 0.25	0.24	0.2–0.3	0.09 ± 0.03	0.10	0.05–0.12

This was the first study in which AME and AOH were detected in pig urine samples (Table 3). Both *Alternaria* toxins were detected frequently (> 50%), which indicates the importance of their determination.

DON was found in 46% of samples with a median concentration of 1.64 ng/mL—similar to other monitoring studies in which DON was found in 57% of samples in concentrations greater than 17.4 ng/mL (Song et al. 2013). The same detection rate for α-ZEL (46%) with a median concentration of 0.54 ng/mL was observed. β-ZEL was only detected rarely (9%) at a concentration lower than the LLOQ value.

In total, 84% (47/56) of the samples were contaminated with one or more mycotoxins. These results demonstrate the applicability of the multi-mycotoxin method to pig urine samples. It is difficult to estimate the exposure of the pigs based solely on an analysis of spot urine samples, because European Union recommendations for mycotoxin levels only exist for pig feed (EC 2006). Nevertheless, some authors have attempted to estimate mycotoxin intake and consequently the level of feed contamination from the urinary mycotoxin and metabolite concentrations (Gambacorta et al. 2019). However, this is only possible if detailed information is obtained concerning mean pig weight, mean daily urine volume and the mean weight of feed consumed daily by the pigs, all of which were not included in our study.

In summary, the LC-MS/MS method based on LLE with EtAc as an extraction solvent for the simultaneous determination of 35 mycotoxins was developed, validated and successfully applied to the analysis of pig urine samples. In comparison with other multi-mycotoxin determination methodologies, this method includes two novel and important steps: optimized enzymatic hydrolysis pretreatment with adjustment to a constant creatinine level. According to the best knowledge of the author, it is the first method used for the determination of nivalenol, citrinin, dihydrocitrinone, fusarenon-X, altertoxin I, tentoxin and hydrolysed fumonisin B_1_ in pig urine samples. Additionally, the matrix effect was assessed for six different pig urine samples, and differences in IS-normalized SSE (lower than 25%) were reported for the first time in pig urine. The method showed a very favourable extraction of polar compounds, such as DON, as well as less polar ones like ZEN. The co-occurrence of alternariol monomethyl ether and alternariol in pig urine is reported herein for the first time. It must be emphasized that for all steps: the LC-MS/MS condition, sample preparation, creatinine adjustment as well as enzymatic hydrolysis are crucial for optimization purposes and provide researchers with a reliable method.

## Supplementary Information

Below is the link to the electronic supplementary material.

Supplementary file1 (DOCX 4 MB)

Supplementary file2 (DOCX 48 KB)

## Data Availability

All data generated or analysed during this study are included in this published article and its supplementary information files.
