# Supplementary material for: Development of a multi-mycotoxin LC-MS/MS method for the determination of biomarkers in pig urine
Source: Mycotoxin Res. 2021 Mar 26;37(2):169–81. doi: 10.1007/s12550-021-00428-w (PMC8163674; doi:10.1007/s12550-021-00428-w)
Supplement: Supplementary file 1 — Supplementary file1 (DOCX 4 MB) [file 12550_2021_428_MOESM1_ESM.docx]

**Supplementary Material:
Development of multi-mycotoxin LC-MS/MS method for determination of biomarkers in pig urine**

**Mycotoxin Research**

**Authors:**

1. **Agnieszka Tkaczyk *** [**https://orcid.org/0000-0003-2929-8077**](https://orcid.org/0000-0003-2929-8077)
2. **Piotr Jedziniak** [**https://orcid.org/0000-0001-5212-2913**](https://orcid.org/0000-0001-5212-2913)

***** Correspondence: [agnieszka.tkaczyk@piwet.pulawy.pl](mailto:agnieszka.tkaczyk@piwet.pulawy.pl)

**Affiliation:**

National Veterinary Research Institute, Department of Pharmacology and Toxicology, Partyzantow 57, 24-100, Pulawy, Poland

**Table S1** MS/MS parameters used for each compound (measured in the ESI negative mode)

| **Analyte** | **Molecular ion** | **Precursor ion  (m/z)** | **Product ions (m/z)** | **Declustering Potential  [V]** | **Collision energy [eV]** | **Retention time  [min]** | **IS** |
| --- | --- | --- | --- | --- | --- | --- | --- |
| **OTα** | [M-H]^-^ | 255 | 167 | -34 | -34 | 5.70 | U-[^13^C_20_]-OTA |
|  |  |  | 211 |  | -24 |  |  |
|  |  |  | 123 |  | -40 |  |  |
| **AOH** | [M-H]^-^ | 257 | 213 | -60 | -30 | 6.83 | U-[^13^C_18_]-ZEN |
|  |  |  | 215 |  | -35 |  |  |
|  |  |  | 147 |  | -43 |  |  |
| **DH-CIT** | [M-H]^-^ | 265 | 221 | -33 | -26 | 5.79 | U-[^13^C_18_]-ZEN |
|  |  |  | 177 |  | -34 |  |  |
|  |  |  | 247 |  | -27 |  |  |
| **AME** | [M-H]^-^ | 271 | 256 | -60 | -31 | 7.46 | U-[^13^C_18_]-ZEN |
|  |  |  | 228 |  | -38 |  |  |
| **CIT** | [M-H+CH_3_OH-]^-^ | 281 | 249 | -60 | -24 | 7.79 | U-[^13^C_18_]-ZEN |
|  |  |  | 205 |  | -31 |  |  |
|  |  |  | 177 |  | -39 |  |  |
| **DONIS** | [M-H]^-^ | 310 | 279 | -60 | -16 | 4.95 | - |
|  |  |  | 228 |  | -25 |  |  |
|  |  |  | 145 |  | -24 |  |  |
| **α-ZEL** | [M-H]^-^ | 319 | 160 | -60 | -38 | 7.33 | U-[^13^C_18_]-ZEN |
|  |  |  | 275 |  | -27 |  |  |
|  |  |  | 174 |  | -34 |  |  |
| **β-ZEL** | [M-H]^-^ | 319 | 160 | -84 | -39 | 6.93 | U-[^13^C_18_]-ZEN |
|  |  |  | 275 |  | -27 |  |  |
|  |  |  | 174 |  | -37 |  |  |
| **ZEN** | [M-H]^-^ | 317 | 175 | -50 | -32 | 7.46 | U-[^13^C_18_]-ZEN |
|  |  |  | 131 |  | -37 |  |  |
|  |  |  | 273 |  | -28 |  |  |
| **β-ZAL** | [M-H]^-^ | 321 | 277 | -60 | -30 | 6.79 | U-[^13^C_18_]-ZEN |
|  |  |  | 303 |  | -29 |  |  |
|  |  |  | 259 |  | -34 |  |  |
| **α-ZAL** | [M-H]^-^ | 321 | 277 | -60 | -30 | 7.20 | U-[^13^C_18_]-ZEN |
|  |  |  | 303 |  | -29 |  |  |
|  |  |  | 259 |  | -34 |  |  |
| **ZAN** | [M-H]^-^ | 319 | 205 | -60 | -30 | 7.36 | U-[^13^C_18_]-ZEN |
|  |  |  | 161 |  | -37 |  |  |
|  |  |  | 275 |  | -28 |  |  |
| **ZEN-IS** | [M-H]^-^ | 335 | 185 | -40 | -33 | 7.46 | - |
|  |  |  | 140 |  | -39 |  |  |
| **ATX-I** | [M-H]^-^ | 351 | 263 | -50 | -43 | 6.61 | U-[^13^C_18_]-ZEN |
|  |  |  | 315 |  | -22 |  |  |
|  |  |  | 305 |  | -30 |  |  |

**Table S1 (continued)** MS/MS parameters used for each compound (measured in the ESI negative mode)

| **Analyte** | **Molecular ion** | **Precursor ion  (m/z)** | **Product ions**  **(m/z)** | **Declustering Potential**  **[V]** | **Collision energy [eV]** | **Retention time**  **[min]** | **IS** |
| --- | --- | --- | --- | --- | --- | --- | --- |
| **DON-IS** | [M+CH_3_COO]^-^ | 370 | 310 | -40 | -14 | 4.95 | - |
|  |  |  | 279 |  | -23 |  |  |
|  |  |  | 261 |  | -23 |  |  |
| **NIV** | [M-H]^-^ | 371 | 281 | -60 | -18 | 4.41 | U-[^13^C_15_]-DON |
|  |  |  | 59 |  | -32 |  |  |
|  |  |  | 203 |  | -30 |  |  |
| **TEN** | [M-H]^-^ | 413 | 141 | -60 | -25 | 6.64 | U-[^13^C_18_]-ZEN |
|  |  |  | 271 |  | -21 |  |  |
| **FUS-X** | [M+CH_3_COO]^-^ | 413 | 263 | -15 | -19 | 5.30 | U-[^13^C_15_]-DON |
|  |  |  | 187 |  | -37 |  |  |

**Table S2** MS/MS parameters used for each compound (measured in the ESI positive mode)

| **Analyte** | **Molecular ion** | | **Precursor ion  (m/z)** | | **Product ions (m/z)** | | **Declustering Potential**  **[V]** | | **Collision energy**  **[eV]** | | **Retention time [min]** | | **IS** | |  |
| --- | --- | --- | --- | --- | --- | --- | --- | --- | --- | --- | --- | --- | --- | --- | --- |
| **DOM-1** | [M+H]^+^ | | 281 | | 233 | | 60 | | 15 | | 5.39 | | U-[^13^C_15_]-DON | |  |
|  |  |  |  |  | 215 | |  |  | 16 | |  |  |  |  |  |
|  |  |  |  |  | 233 | |  |  | 15 | |  |  |  |  |  |
| **DON** | [M+H]^+^ | | 297 | | 249 | | 9 | | 16 | | 4.95 | | U-[^13^C_15_]-DON | |  |
|  |  |  |  |  | 203 | |  |  | 21 | |  |  |  |  |  |
| **AFB_1_** | [M+H]^+^ | | 313 | | 241 | | 56 | | 49 | | 6.37 | | U-[^13^C_17_]-AFLB_1_ | |  |
|  |  |  |  |  | 285 | |  |  | 32 | |  |  |  |  |  |
|  |  |  |  |  | 269 | |  |  | 43 | |  |  |  |  |  |
| **AFB_2_** | [M+H]^+^ | | 315 | | 259 | | 50 | | 39 | | 6.26 | | U-[^13^C_17_]-AFLB_1_ | |  |
|  |  |  |  |  | 287 | |  |  | 34 | |  |  |  |  |  |
|  |  |  |  |  | 243 | |  |  | 51 | |  |  |  |  |  |
| **STC** | [M+H]^+^ | | 325 | | 281 | | 60 | | 48 | | 7.80 | | U-[^13^C_17_]-AFLB_1_ | |  |
|  |  |  |  |  | 310 | |  |  | 34 | |  |  |  |  |  |
|  |  |  |  |  | 253 | |  |  | 50 | |  |  |  |  |  |
| **AFM_1_** | [M+H]^+^ | | 329 | | 273 | | 40 | | 48 | | 5.97 | | U-[^13^C_17_]-AFLB_1_ | |  |
|  |  |  |  |  | 229 | |  |  | 42 | |  |  |  |  |  |
| **AFG_1_** | [M+H]^+^ | | 329 | | 200 | | 40 | | 53 | | 6.37 | | U-[^13^C_17_]-AFLB_1_ | |  |
|  |  |  |  |  | 243 | |  |  | 35 | |  |  |  |  |  |
| **AFB_1_ IS** | [M+H]^+^ | | 330 | | 301 | | 40 | | 32 | | 6.37 | | - | |  |
|  |  |  |  |  | 255 | |  |  | 49 | |  |  |  |  |  |
| **15-AcDON** | [M+H]^+^ | | 339 | | 261 | | 31 | | 15 | | 6.73 | | U-[^13^C_15_]-DON | |  |
|  |  |  |  |  | 321 | |  |  | 12 | |  |  |  |  |  |
|  |  |  |  |  | 137 | |  |  | 14 | |  |  |  |  |  |
| **3-AcDON** | [M+H]^+^ | | 339 | | 231 | | 40 | | 17 | | 6.76 | | U-[^13^C_15_]-DON | |  |
|  |  |  |  |  | 213 | |  |  | 23 | |  |  |  |  |  |
|  |  |  |  |  | 175 | |  |  | 31 | |  |  |  |  |  |
| **DAS** | [M+NH_4_]^+^ | | 384 | | 307 | | 60 | | 13 | | 6.31 | | U-[^13^C_20_]-OTA | |  |
|  |  |  |  |  | 247 | |  |  | 18 | |  |  |  |  |  |
|  |  |  |  |  | 105 | |  |  | 60 | |  |  |  |  |  |
| **T-2 triol** | [M+NH_4_]^+^ | | 400 | | 215 | | 40 | | 16 | | 6.31 | | U-[^13^C_24_]-T-2 | |  |
|  |  |  |  |  | 281 | |  |  | 13 | |  |  |  |  |  |
|  |  |  |  |  | 233 | |  |  | 11 | |  |  |  |  |  |
| **OTA** | [M+H]^+^ | | 404 | | 239 | | 40 | | 34 | | 6.59 | | U-[^13^C_20_]-OTA | |  |
|  |  |  |  |  | 358 | |  |  | 18 | |  |  |  |  |  |
|  |  |  |  |  | 102 | |  |  | 89 | |  |  |  |  |  |
| **HFB_1_** | [M+H]^+^ | | 406 | | 388 | | 40 | | 24 | | 6.52 | | U-[^13^C_20_]-OTA | |  |
|  |  |  |  |  | 370 | |  |  | 26 | |  |  |  |  |  |
|  |  |  |  |  | 352 | |  |  | 30 | |  |  |  |  |  |
| **OTA IS** | | [M+H]^+^ | | 424 | | 250 | | 40 | | 33 | | 6.59 | | - | |
|  |  |  |  |  |  | 232 | |  |  | 50 | |  |  |  |  |
|  |  |  |  |  |  | 203 | |  |  | 58 | |  |  |  |  |

**Table S2 (continued)** MS/MS parameters used for each compound (measured in the ESI positive mode)

| **Analyte** | **Molecular ion** | **Precursor ion  (m/z)** | **Product ions (m/z)** | **Declustering Potential**  **[V]** | **Collision energy (eV)** | **Retention time**  **[min]** | **IS** |
| --- | --- | --- | --- | --- | --- | --- | --- |
| **HT-2** | [M+NH_4_]^+^ | 442 | 215 | 17 | 17 | 6.64 | U-[^13^C_24_]-T-2 |
|  |  |  | 263 |  | 17 |  |  |
|  |  |  | 115 |  | 132 |  |  |
| **T-2** | [M+Na]^+^ | 489 | 245 | 73 | 37 | 7.02 | U-[^13^C_24_]-T-2 |
|  |  |  | 327 |  | 30 |  |  |
|  |  |  | 387 |  | 31 |  |  |
| **T-2 IS** | [M+NH4]+ | 508 | 322 | 40 | 19 | 7.02 | - |
|  |  |  | 229 |  | 26 |  |  |
|  |  |  | 198 |  | 30 |  |  |
| **ENB** | [M+H]^+^ | 640 | 196 | 90 | 30 | 9.30 | U-[^13^C_18_]-ZEN |
|  |  |  | 214 |  | 35 |  |  |
|  |  |  | 186 |  | 47 |  |  |
| **ENB_1_** | [M+H]^+^ | 654 | 196 | 40 | 33 | 9.51 | U-[^13^C_18_]-ZEN |
|  |  |  | 210 |  | 31 |  |  |
|  |  |  | 214 |  | 33 |  |  |
| **ENA_1_** | [M+H]^+^ | 668 | 210 | 97 | 28 | 9.74 | U-[^13^C_18_]-ZEN |
|  |  |  | 228 |  | 31 |  |  |
|  |  |  | 196 |  | 31 |  |  |
| **ENA** | [M+H]^+^ | 699 | 210 | 48 | 39 | 9.97 | U-[^13^C_18_]-ZEN |
|  |  |  | 228 |  | 39 |  |  |
|  |  |  | 328 |  | 53 |  |  |
| **BEA** | [M+H]^+^ | 784 | 244 | 60 | 34 | 9.45 | U-[^13^C_18_]-ZEN |
|  |  |  | 134 |  | 84 |  |  |
|  |  |  | 119 |  | 155 |  |  |

**Table S3** Concentration of analytes in quality control (QC) samples **(spiked urine)**

| **Analyte/ level [ng/mL]** | **LLOQ** | **low QC** | **medium QC** | **high QC** |
| --- | --- | --- | --- | --- |
| **CIT** | 0.5 | 1 | 4 | 10 |
| **α-ZEL** | 0.4 | 0.8 | 3.2 | 8 |
| **β-ZEL** | 0.6 | 1.2 | 4.8 | 12 |
| **ZEN** | 0.1 | 0.2 | 0.8 | 2 |
| **β-ZAL** | 3 | 6 | 24 | 60 |
| **α-ZAL** | 2 | 4 | 16 | 40 |
| **ZAN** | 0.5 | 1 | 4 | 10 |
| **NIV** | 8 | 16 | 64 | 160 |
| **FUS-X** | 2 | 4 | 16 | 40 |
| **DOM-1** | 6 | 12 | 48 | 120 |
| **DON** | 2 | 4 | 16 | 20 |
| **AFB_1_** | 0.5 | 1 | 4 | 10 |
| **AFB_2_** | 0.5 | 1 | 4 | 10 |
| **STC** | 0.1 | 0.2 | 0.8 | 2 |
| **AFM_1_** | 0.5 | 1 | 4 | 10 |
| **AFG_1_** | 0.5 | 1 | 4 | 10 |
| **15-AcDON** | 8 | 16 | 64 | 80 |
| **3-AcDON** | 2 | 4 | 16 | 40 |
| **DAS** | 0.5 | 1 | 4 | 10 |
| **OTA** | 1.5 | 3 | 12 | 30 |
| **HT-2** | 1.5 | 3 | 12 | 30 |
| **T-2** | 1 | 2 | 8 | 20 |
| **ENB** | 0.2 | 0.4 | 1.6 | 4 |
| **ENB_1_** | 0.2 | 0.4 | 1.6 | 4 |
| **ENA_1_** | 0.2 | 0.4 | 1.6 | 4 |
| **ENA** | 0.2 | 0.4 | 1.6 | 4 |
| **BEA** | 0.2 | 0.4 | 1.6 | 4 |
| **AOH** | 1 | 2 | 8 | 20 |
| **AME** | 0.2 | 0.4 | 1.6 | 4 |
| **ATX-I** | 0.5 | 1 | 4 | 10 |
| **TEN** | 0.4 | 0.8 | 3.2 | 8 |
| **OTα** | 4 | 8 | 32 | 80 |
| **DH-CIT** | 4 | 8 | 32 | 80 |
| **T-2 triol** | 2 | 4 | 16 | 40 |
| **HFB_1_** | 4 | 8 | 32 | 80 |

**Table S4** Values of accuracy and precision

1. Within-Day accuracy and precision (n=6)

| **Analyte** | **Theoretical Concentration  LLOQ** | | **Theoretical Concentration  low QC** | | **Theoretical Concentration  medium QC** | | **Theoretical Concentration  high QC** | |
| --- | --- | --- | --- | --- | --- | --- | --- | --- |
|  | **Accuracy (%)** | **Precision (RSD %)** | **Accuracy (%)** | **Precision (RSD %)** | **Accuracy (%)** | **Precision (RSD %)** | **Accuracy (%)** | **Precision (RSD %)** |
| **CIT** | 117 | 3.38 | 107 | 0.03 | 105 | 11.51 | 110 | 8.22 |
| **α-ZEL** | 88.6 | 7.88 | 97.5 | 4.66 | 101 | 8.92 | 91.8 | 4.72 |
| **β-ZEL** | 97.1 | 7.00 | 97.9 | 9.64 | 94.4 | 11.95 | 97.7 | 9.83 |
| **ZEN** | 101 | 5.07 | 104 | 3.37 | 99.3 | 7.32 | 92.8 | 1.76 |
| **β-ZAL** | 91.2 | 8.76 | 99.9 | 7.97 | 95.0 | 10.38 | 73.7 | 5.87 |
| **α-ZAL** | 81.7 | 13.87 | 97.7 | 5.53 | 102 | 4.36 | 91.9 | 5.15 |
| **ZAN** | 85.2 | 17.22 | 103 | 4.81 | 104 | 8.44 | 95.2 | 5.18 |
| **NIV** | 100 | 11.35 | 87.2 | 8.24 | 88.5 | 5.59 | 91.6 | 7.45 |
| **FUS-X** | 101 | 10.53 | 102 | 8.28 | 105 | 6.30 | 102 | 4.64 |
| **DOM-1** | 108 | 8.79 | 106 | 4.85 | 105 | 4.32 | 102 | 6.93 |
| **DON** | 103 | 10.38 | 104 | 7.52 | 105 | 6.22 | 103 | 6.13 |
| **AFB_1_** | 102 | 15.97 | 98.0 | 5.48 | 103 | 4.97 | 98.5 | 5.81 |
| **AFB_2_** | 96.0 | 8.85 | 101 | 5.73 | 102 | 4.15 | 92.7 | 5.97 |
| **STC** | 98.0 | 12.38 | 103 | 8.84 | 109 | 2.78 | 92.5 | 5.91 |
| **AFLM_1_** | 96.0 | 4.85 | 101 | 8.05 | 103 | 5.83 | 92.7 | 4.63 |
| **AFG_1_** | 101 | 8.34 | 107 | 3.32 | 104 | 7.54 | 99.4 | 5.39 |
| **15-AcDON** | 103 | 5.00 | 106 | 4.73 | 109 | 5.02 | 103 | 7.79 |
| **3-AcDON** | 103 | 7.45 | 104 | 5.08 | 110 | 2.79 | 104 | 7.24 |
| **DAS** | 85.2 | 9.97 | 103 | 8.63 | 105 | 6.61 | 87.9 | 5.87 |
| **OTA** | 105 | 7.76 | 102 | 7.26 | 96.7 | 5.95 | 98.8 | 4.37 |
| **HT-2** | 89.4 | 10.03 | 101 | 8.90 | 105 | 6.21 | 91.4 | 7.59 |
| **T-2** | 87.1 | 11.21 | 95.6 | 6.37 | 90.8 | 4.30 | 93.2 | 4.85 |
| **ENB** | 107 | 5.25 | 99.2 | 4.46 | 104 | 4.18 | 102 | 3.65 |
| **ENB_1_** | 111 | 5.36 | 75.6 | 5.15 | 98.0 | 6.52 | 103 | 9.49 |
| **ENA_1_** | 116 | 2.47 | 73.1 | 10.09 | 87.5 | 8.30 | 109 | 2.64 |
| **ENA** | 100 | 16.06 | 67.3 | 14.57 | 58.7 | 13.78 | 87.0 | 7.11 |
| **BEA** | 125 | 10.42 | 66.6 | 12.80 | 74.0 | 14.06 | 118 | 3.67 |
| **AOH** | 92.0 | 8.99 | 101 | 5.26 | 102 | 9.50 | 90.3 | 9.57 |
| **AME** | 92.7 | 8.90 | 108 | 3.92 | 112 | 1.00 | 98.8 | 5.41 |
| **ATX-I** | 92.7 | 7.71 | 89.9 | 4.84 | 84.0 | 9.89 | 88.1 | 12.76 |
| **TEN** | 77.5 | 10.30 | 95.1 | 8.26 | 96.1 | 15.59 | 82.4 | 18.15 |
| **OTα** | 85.8 | 6.76 | 104 | 10.65 | 108 | 5.77 | 110 | 15.30 |
| **HFB_1_** | 112 | 11.23 | 70.3 | 13.20 | 111 | 5.09 | 108 | 16.37 |
| **DH-CIT** | 97.1 | 7.63 | 99.6 | 3.77 | 107 | 3.91 | 94.2 | 5.00 |
| **T-2 triol** | 98.8 | 8.35 | 105 | 4.99 | 102 | 7.75 | 90.4 | 11.91 |

**Table S4** (continued) Values of accuracy and precision

1. Between-Day accuracy and precision (n=6)

| **Analyte** | **Theoretical Concentration**  **LLOQ** | | **Theoretical Concentration**  **low QC** | | **Theoretical Concentration**  **medium QC** | | **Theoretical Concentration**  **high QC** | |
| --- | --- | --- | --- | --- | --- | --- | --- | --- |
|  | **Accuracy (%)** | **Precision (RSD %)** | **Accuracy (%)** | **Precision (RSD %)** | **Accuracy (%)** | **Precision (RSD %)** | **Accuracy (%)** | **Precision (RSD %)** |
| **CIT** | 112 | 10.79 | 96.0 | 14.68 | 92.2 | 17.89 | 107 | 7.01 |
| **α-ZEL** | 95.2 | 11.25 | 102 | 7.40 | 104 | 7.01 | 97.9 | 8.09 |
| **β-ZEL** | 102 | 9.05 | 99.6 | 8.67 | 96.1 | 12.25 | 102 | 8.14 |
| **ZEN** | 98.2 | 5.68 | 99.6 | 4.84 | 104 | 6.13 | 98.1 | 7.74 |
| **β-ZAL** | 97.1 | 9.18 | 103 | 7.71 | 100 | 10.35 | 93.9 | 9.21 |
| **α-ZAL** | 94.4 | 11.96 | 104 | 5.90 | 104 | 6.69 | 97.9 | 8.12 |
| **ZAN** | 96.2 | 13.21 | 102 | 6.28 | 103 | 12.60 | 98.5 | 7.50 |
| **NIV** | 105 | 13.03 | 93.6 | 13.56 | 98.5 | 13.43 | 103 | 11.16 |
| **FUS-X** | 93.7 | 10.94 | 101 | 10.90 | 101 | 13.92 | 100 | 12.30 |
| **DOM-1** | 102 | 11.83 | 99.9 | 9.72 | 98.6 | 16.90 | 100 | 13.61 |
| **DON** | 98.5 | 8.82 | 103 | 7.24 | 100 | 13.76 | 101 | 9.60 |
| **AFB_1_** | 100.0 | 13.91 | 97.8 | 5.87 | 102 | 10.38 | 98.4 | 7.69 |
| **AFB_2_** | 98.7 | 9.09 | 99.6 | 5.28 | 99.5 | 15.61 | 98.6 | 11.76 |
| **STC** | 94.3 | 13.80 | 103 | 7.72 | 104 | 10.63 | 97.2 | 15.12 |
| **AFLM_1_** | 94.3 | 11.93 | 103 | 6.35 | 103 | 14.12 | 97.0 | 13.59 |
| **AFG_1_** | 99.8 | 13.29 | 99.4 | 13.04 | 102 | 10.39 | 99.1 | 7.92 |
| **15-AcDON** | 100 | 11.29 | 100 | 14.78 | 102 | 19.42 | 100 | 12.84 |
| **3-AcDON** | 100 | 10.54 | 101 | 9.96 | 101 | 17.99 | 101 | 12.80 |
| **DAS** | 94.5 | 11.93 | 103 | 8.24 | 105 | 7.07 | 97.4 | 9.50 |
| **OTA** | 96.0 | 7.54 | 103 | 4.63 | 103 | 6.17 | 97.8 | 4.95 |
| **HT-2** | 94.5 | 10.65 | 104 | 7.37 | 104 | 8.58 | 97.9 | 7.89 |
| **T-2** | 96.3 | 12.95 | 104 | 7.99 | 99.6 | 9.40 | 99.7 | 8.46 |
| **ENB** | 103 | 8.93 | 97.3 | 8.64 | 100 | 7.03 | 89.3 | 5.71 |
| **ENB_1_** | 111 | 6.26 | 87.1 | 8.30 | 93.2 | 7.06 | 92.6 | 5.96 |
| **ENA_1_** | 110 | 4.16 | 85.0 | 14.48 | 82.8 | 10.76 | 97.7 | 4.03 |
| **ENA** | 110 | 26.43 | 81.5 | 26.82 | 67.5 | 25.30 | 93.3 | 6.48 |
| **BEA** | 125 | 14.22 | 79.6 | 21.19 | 75.4 | 18.77 | 108 | 25.85 |
| **AOH** | 94.8 | 12.83 | 104 | 6.99 | 104 | 8.16 | 98.2 | 9.58 |
| **AME** | 93.6 | 15.57 | 105 | 9.27 | 111 | 6.56 | 98.0 | 7.83 |
| **ATX-I** | 103 | 10.10 | 99.9 | 10.28 | 93.5 | 14.41 | 103 | 12.46 |
| **TEN** | 93.7 | 15.86 | 101 | 6.36 | 106 | 18.96 | 95.5 | 20.36 |
| **OTα** | 93.2 | 16.58 | 103 | 7.20 | 105 | 6.87 | 106 | 11.92 |
| **HFB_1_** | 105 | 25.06 | 70.4 | 14.89 | 75.4 | 9.04 | 72.6 | 13.94 |
| **DH-CIT** | 96.6 | 7.02 | 101 | 7.51 | 105 | 7.43 | 97.9 | 8.62 |
| **T-2 triol** | 97.1 | 9.12 | 103 | 7.21 | 100 | 11.76 | 99.6 | 10.75 |

**Table S4 (continued)** Values of accuracy and precision

1. Two different batch of samples – accuracy and precision (n=6)

| **Analyte** | **Theoretical Concentration**  **LLOQ** | | **Theoretical Concentration**  **low QC** | | **Theoretical Concentration**  **medium QC** | | **Theoretical Concentration**  **high QC** | |
| --- | --- | --- | --- | --- | --- | --- | --- | --- |
|  | **Accuracy (%)** | **Precision (RSD %)** | **Accuracy (%)** | **Precision (RSD %)** | **Accuracy (%)** | **Precision (RSD %)** | **Accuracy (%)** | **Precision (RSD %)** |
| **CIT** | 104 | 8.66 | 101 | 7.75 | 92.2 | 12.70 | 103 | 9.36 |
| **α-ZEL** | 95.3 | 10.01 | 104 | 5.14 | 102 | 4.31 | 97.7 | 4.80 |
| **β-ZEL** | 105 | 8.33 | 99.0 | 9.77 | 93.2 | 9.22 | 102 | 10.50 |
| **ZEN** | 96.3 | 4.67 | 103 | 6.59 | 102 | 4.54 | 98.2 | 5.24 |
| **β-ZAL** | 98.8 | 8.96 | 102 | 8.46 | 99.3 | 7.59 | 99.0 | 9.21 |
| **α-ZAL** | 97.0 | 10.14 | 103 | 9.37 | 99.6 | 6.87 | 98.8 | 7.23 |
| **ZAN** | 95.7 | 6.08 | 103 | 5.35 | 103 | 6.38 | 97.2 | 5.65 |
| **NIV** | 111 | 13.39 | 91.2 | 14.30 | 94.9 | 12.03 | 102 | 9.29 |
| **FUS-X** | 97.6 | 16.15 | 104 | 11.42 | 98.3 | 10.33 | 102 | 9.61 |
| **DOM-1** | 104 | 11.06 | 98.0 | 11.56 | 95.4 | 12.26 | 104 | 7.89 |
| **DON** | 103 | 6.62 | 97.9 | 7.85 | 98.2 | 8.72 | 101 | 7.24 |
| **AFB_1_** | 98.3 | 6.62 | 102 | 12.86 | 99.8 | 8.30 | 98.7 | 10.49 |
| **AFB_2_** | 94.3 | 5.63 | 107 | 3.92 | 98.0 | 9.98 | 99.9 | 8.43 |
| **STC** | 101 | 8.59 | 99.9 | 5.28 | 98.7 | 7.74 | 101 | 5.17 |
| **AFLM_1_** | 90.3 | 12.53 | 109 | 5.06 | 103 | 9.50 | 97.8 | 8.63 |
| **AFG_1_** | 92.9 | 9.96 | 108 | 7.46 | 99.8 | 9.60 | 100 | 10.03 |
| **15-AcDON** | 103 | 10.16 | 99.4 | 9.51 | 94.8 | 9.09 | 104 | 8.12 |
| **3-AcDON** | 104 | 8.68 | 99.0 | 8.98 | 95.0 | 11.90 | 103 | 7.35 |
| **DAS** | 94.2 | 8.40 | 104 | 7.35 | 105 | 6.71 | 96.4 | 6.19 |
| **OTA** | 90.1 | 4.62 | 107 | 4.35 | 107 | 5.57 | 94.5 | 6.67 |
| **HT-2** | 95.8 | 12.00 | 102 | 6.11 | 105 | 4.51 | 97.3 | 5.69 |
| **T-2** | 98.8 | 5.71 | 99.4 | 9.05 | 103 | 6.43 | 98.7 | 5.57 |
| **ENB** | 109 | 12.41 | 92.7 | 7.44 | 95.5 | 8.57 | 104 | 6.91 |
| **ENB_1_** | 115 | 10.57 | 89.3 | 9.13 | 90.4 | 11.52 | 107 | 6.90 |
| **ENA_1_** | 118 | 4.52 | 89.4 | 13.08 | 85.0 | 18.95 | 110 | 4.35 |
| **ENA** | 129 | 15.80 | 84.7 | 14.39 | 72.3 | 28.33 | 119 | 13.22 |
| **BEA** | 127 | 11.47 | 85.1 | 15.26 | 74.9 | 19.37 | 116 | 6.93 |
| **AOH** | 96.1 | 10.12 | 104 | 4.49 | 100 | 5.76 | 98.9 | 5.07 |
| **AME** | 95.3 | 9.26 | 103 | 7.57 | 104 | 6.56 | 97.6 | 2.97 |
| **ATX-I** | 106 | 8.02 | 99.7 | 8.31 | 89.9 | 4.97 | 104 | 9.38 |
| **TEN** | 94.0 | 7.30 | 103 | 6.85 | 105 | 5.60 | 97.2 | 7.25 |
| **OTα** | 102 | 4.41 | 98.0 | 6.42 | 99.4 | 5.36 | 101 | 5.28 |
| **HFB_1_** | 105 | 6.66 | 97.1 | 9.99 | 95.7 | 9.63 | 102 | 5.86 |
| **DH-CIT** | 101 | 4.40 | 103 | 3.65 | 94.1 | 5.69 | 101 | 12.20 |
| **T-2 triol** | 93.4 | 9.46 | 105 | 6.36 | 104 | 9.98 | 97.2 | 8.64 |

**
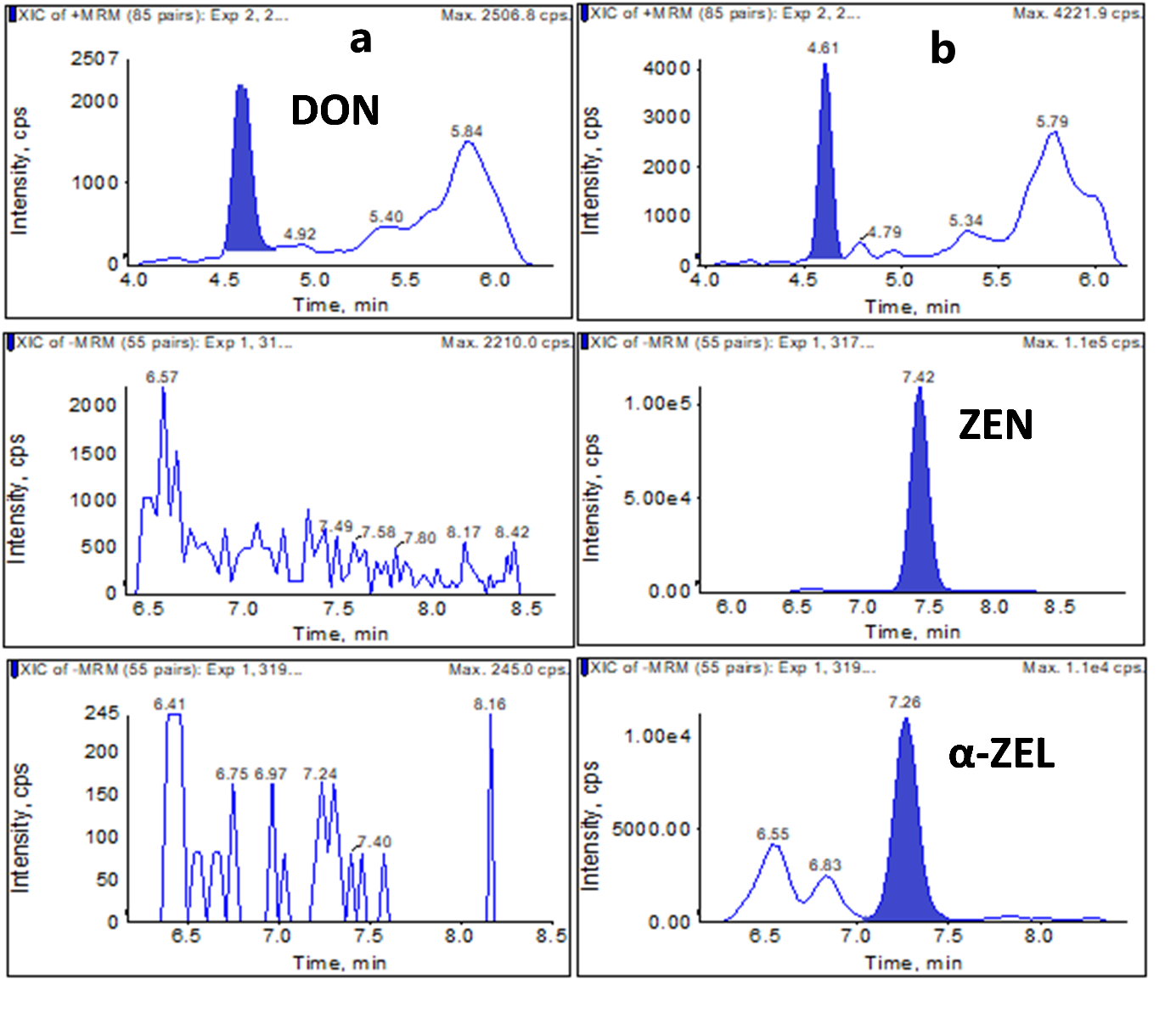
Fig. S1** Chromatogram of DON, ZEN, α-ZEL extracted from pig urine sample a) before and b) after enzymatic hydrolysis (beta-glucuronidase from E.coli)


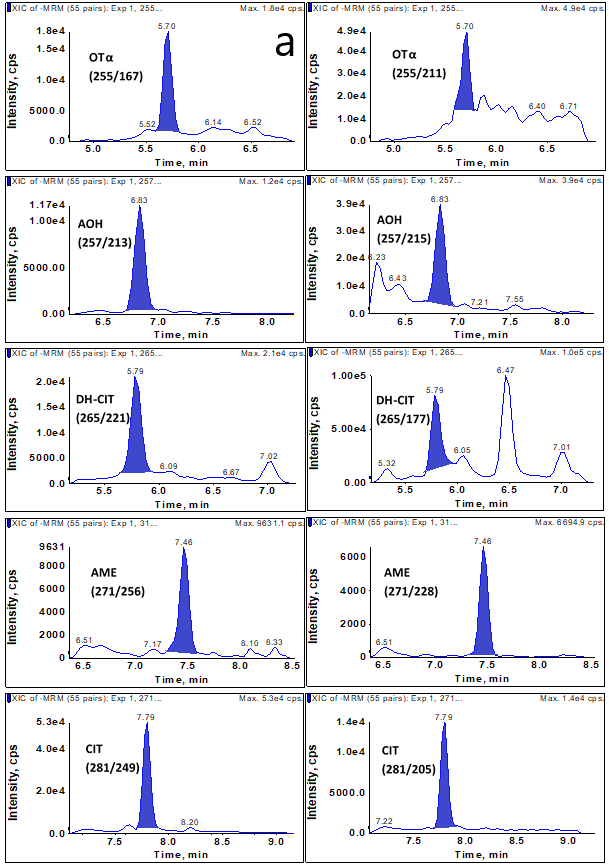


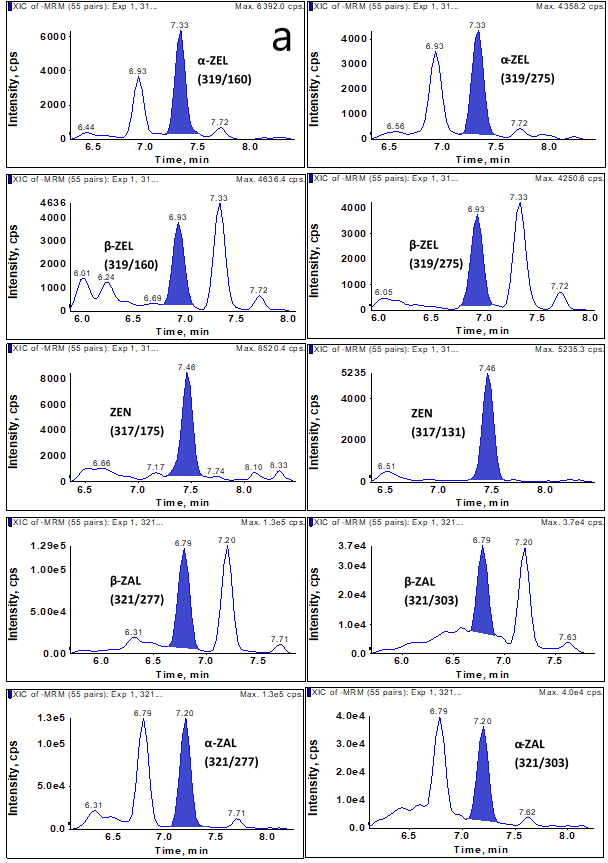


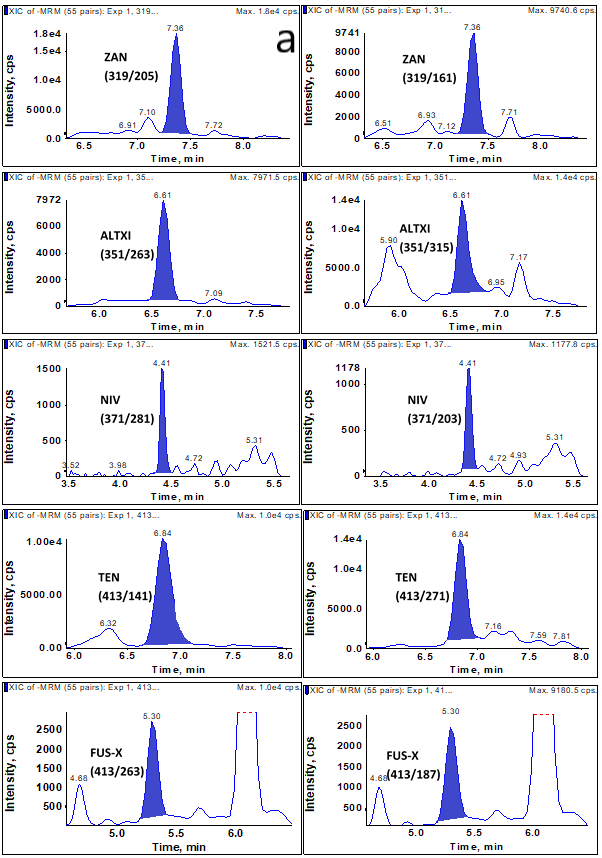


**
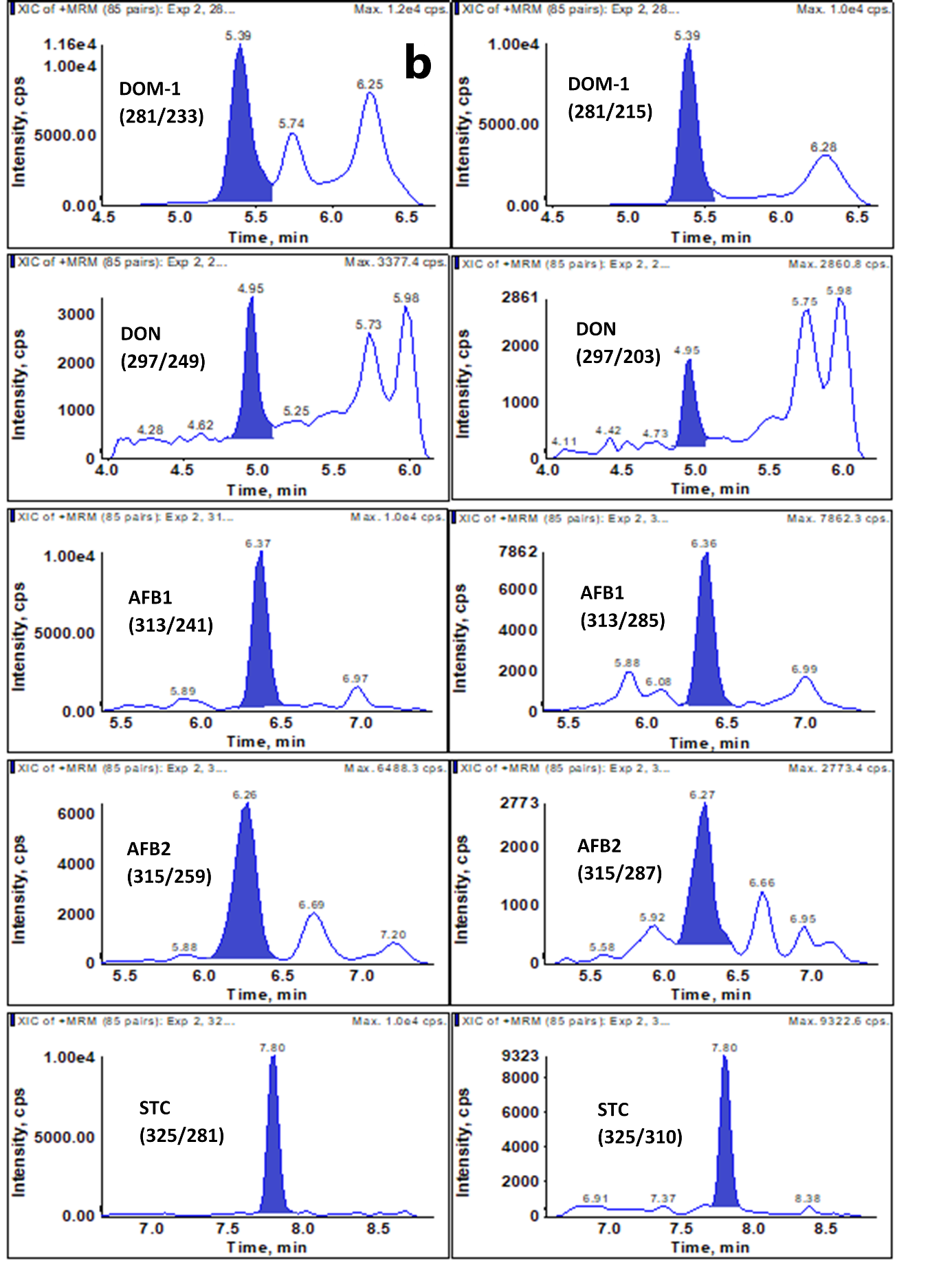
**

**
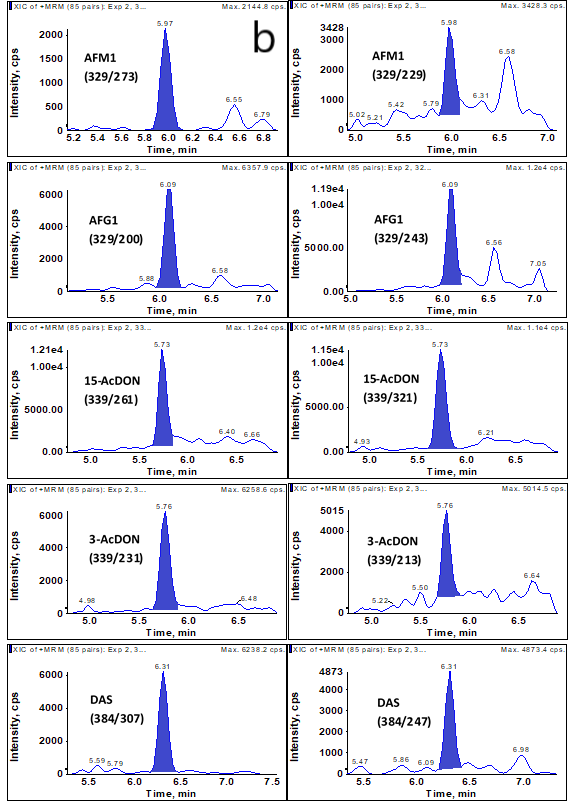
**

**
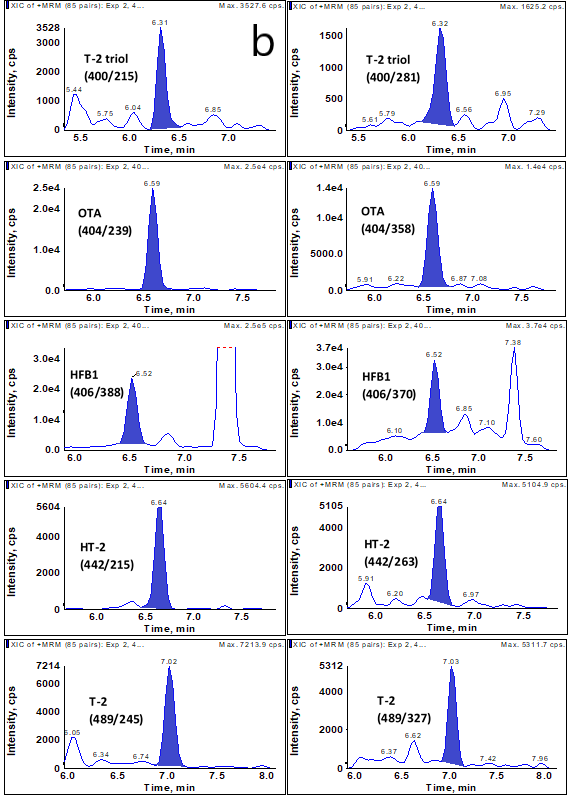
**


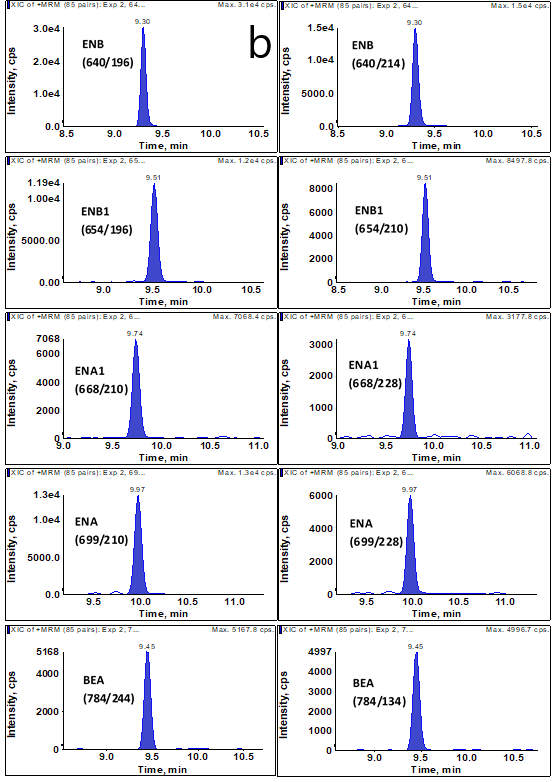


**Fig. S2** Chromatogram (transitions used for quantification and confirmation) obtained from

pig urine sample spiked with (level I) mycotoxins: a) Ion chromatogram of mycotoxins mixture

(negative ionization), b) Ion chromatogram of mycotoxins mixture (positive ionization).


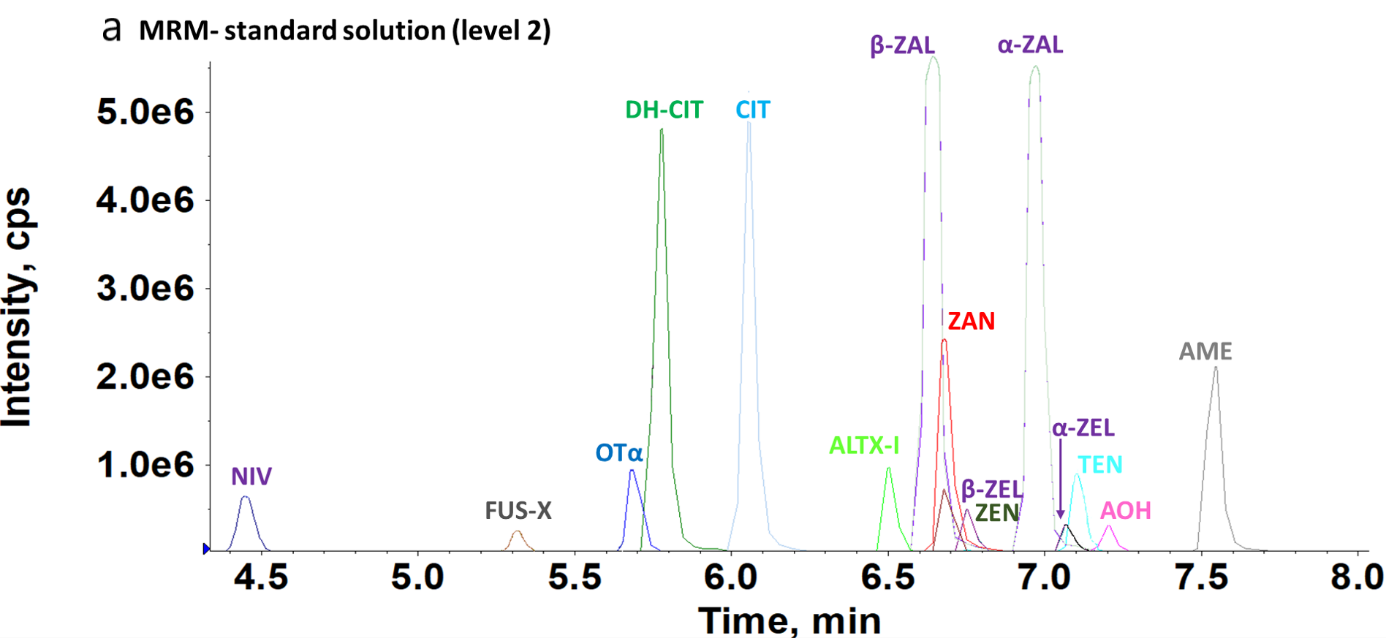


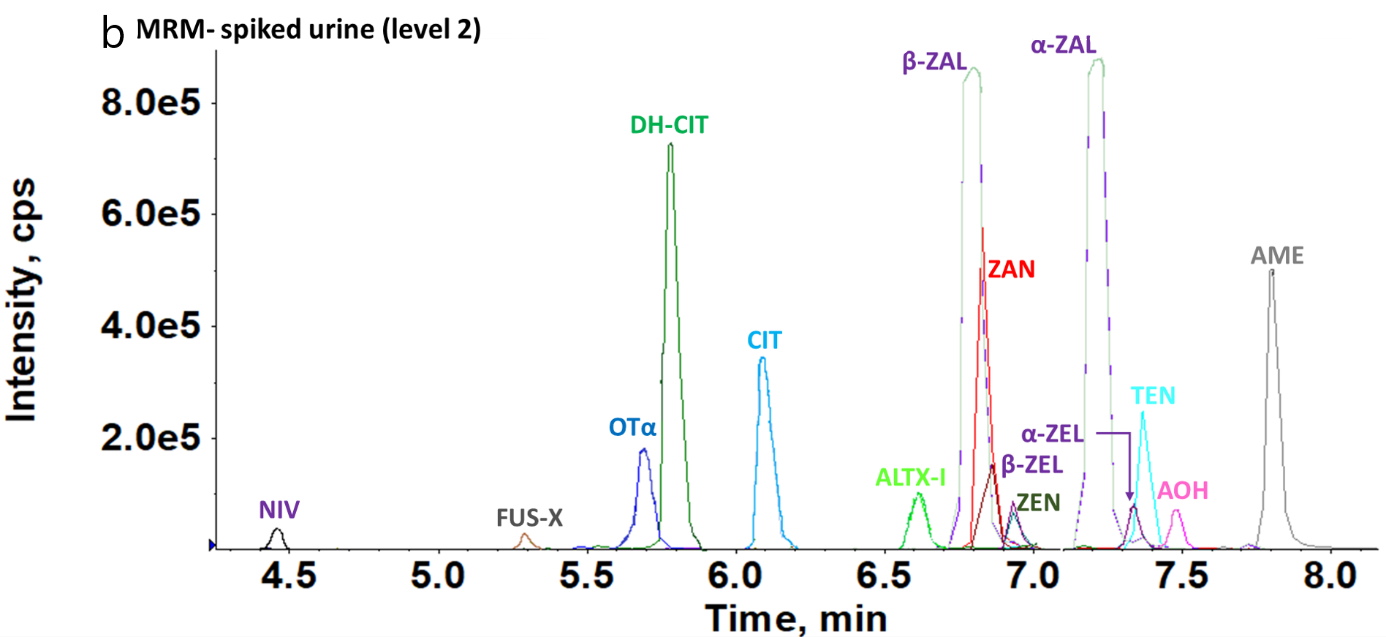


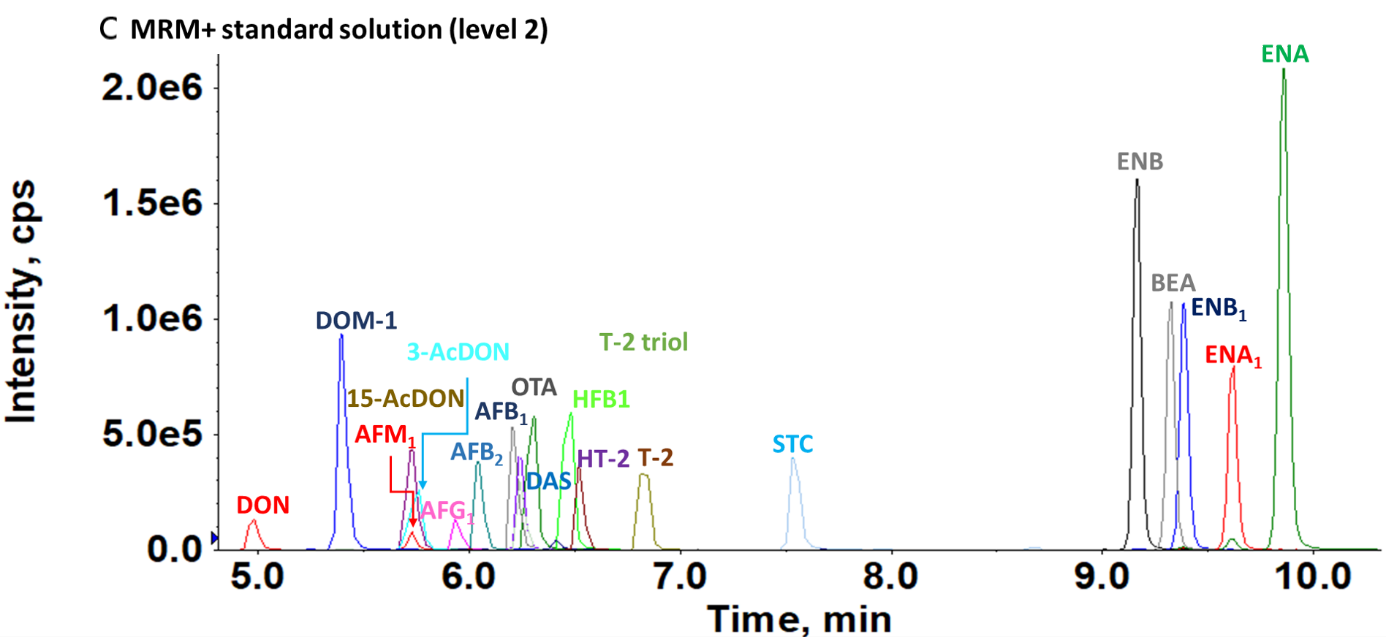


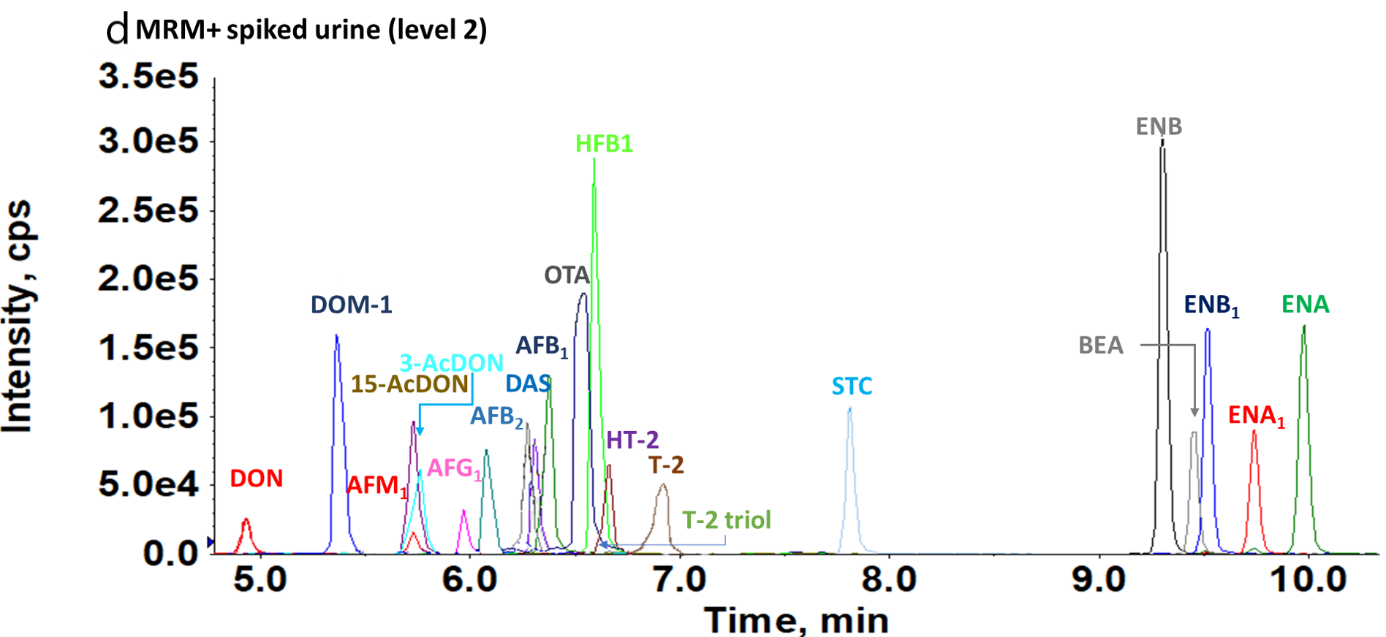


**Fig. S3** Ion chromatogram of mycotoxins mixture (level 2):

1. Standard solution – negative ionisation
2. Pig urine sample spiked with mycotoxins - negative ionization
3. Standard solution – positive ionisation
4. Pig urine sample spiked with mycotoxins - positive ionization

**
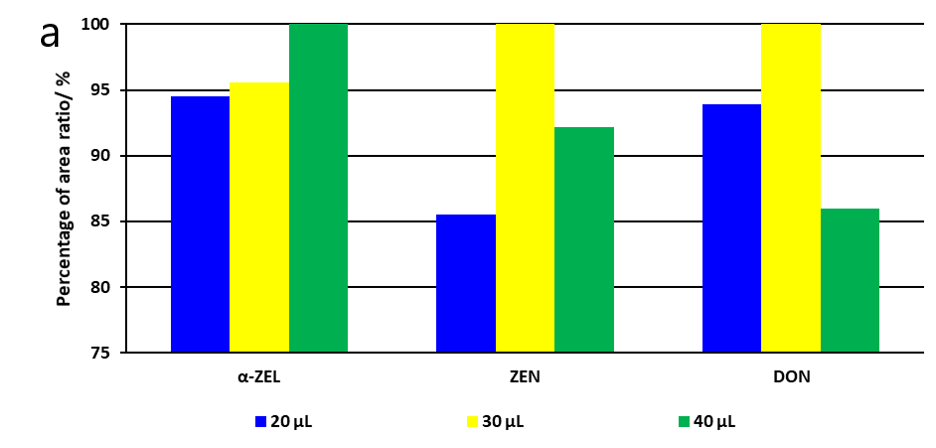
**

**
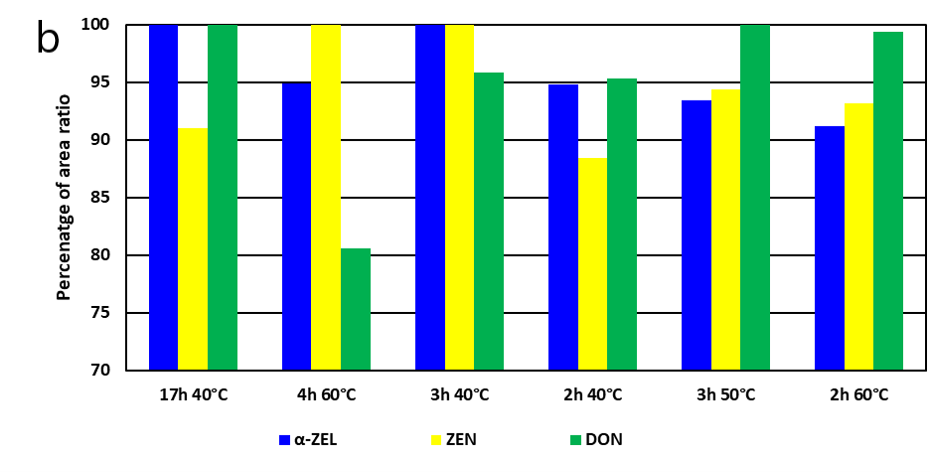
**

**Fig. S4** Comparison of percentage of area ratio of a) enzyme (beta-glucuronidase from E.coli) volume, b) hydrolysis time and temperature of urine sample contaminated with α-ZEL, ZEN and DON.

**

**

**Fig. S5** Comparison of extraction recovery (R_E_) of EtAc with acidified EtAc (1% HCOOH), EtAc:MeOH (7.5:5) and two steps extraction: 2xEtAc and EtAc with acidified EtAc(1% HCOOH)





**Fig. S6** Comparison of extraction recovery for selected mycotoxins after addition of different salts.

**

**

**Fig. S7** Impact of different pH on peak area of selected mycotoxins





**Fig. S8** Matrix effect performed by LLE, IAC and SPE





**Fig. S9** S/N values performed by LLE, IAC and SPE
