# Supplementary material for: Development of a multi-mycotoxin LC-MS/MS method for the determination of biomarkers in pig urine
Source: Mycotoxin Res. 2021 Mar 26;37(2):169–81. doi: 10.1007/s12550-021-00428-w (PMC8163674; doi:10.1007/s12550-021-00428-w)
Supplement: Supplementary file 2 — Supplementary file2 (DOCX 48 KB) [file 12550_2021_428_MOESM2_ESM.docx]

**Supplementary Material:
Development of multi-mycotoxin LC-MS/MS method for determination of biomarkers in pig urine**

**Mycotoxin Research**

**Authors:**

1. Agnieszka Tkaczyk * <https://orcid.org/0000-0003-2929-8077>
2. Piotr Jedziniak <https://orcid.org/0000-0001-5212-2913>

***** Correspondence: [agnieszka.tkaczyk@piwet.pulawy.pl](mailto:agnieszka.tkaczyk@piwet.pulawy.pl)

**Affiliation:**

National Veterinary Research Institute, Department of Pharmacology and Toxicology, Partyzantow 57, 24-100, Pulawy, Poland

**Table S1a** Summary of recent sample preparation for LC-MS/MS analysis of mycotoxin biomarkers in pig urine samples.

| **Sample preparation** | | **Creatinine adjustment** | **Enzymatic hydrolysis (enzyme)** | **Analyte** | **LOD [ng/mL]** | **LOQ [ng/mL]** | **Recovery [%]** | **Matrix effect (SSE)  [%]** | **LC-MS** | **References** |
| --- | --- | --- | --- | --- | --- | --- | --- | --- | --- | --- |
| **Solid Phase Extraction (SPE) columns** | RP-18 SPE (Phenomenex) | - | glucuronidase/ arylsulfatase from Helix pomatia | ZEN  α-ZEL  β-ZEL  α-ZAL  β-ZAL | 0.1  0.2  0.2  0.5  0.5 | 0.5  0.5  0.5  1  1 | 94-105 | - | PE Sciex API 365  (APCI) | (Jodlbauer et al. 2000) |
|  | Oasis™ HLB columns (Waters) | - | type H-2 from Helix pomatia | ZEN  α-ZEL  β-ZEL  ZAN  α-ZAL  β-ZAL  DON  DOM-1 | 0.03  0.07  0.16  0.09  0.11  0.13  0.11  0.04 | 0.10  0.24  0.52  0.30  0.35  0.44  0.38  0.15 | 76-118 | - | 1200 series HPLC system (Agilent Technologies) coupled to a 4000 QTrap (Applied Biosystems) | (Brezina et al. 2014) |

| **Sample preparation** | **Creatinine adjustment** | **Enzymatic hydrolysis (enzyme)** | **Analyte** | **LOD [ng/mL]** | **LOQ [ng/mL]** | **Recovery [%]** | **Matrix effect (SSE)  [%]** | **LC-MS** | **References** |
| --- | --- | --- | --- | --- | --- | --- | --- | --- | --- |
| **Myco6in1** immunoaffinity column (IAC) (Vicam) and an OASIS HLB solid phase extraction **(SPE**) column (Waters) connected in tandem | - | β-glucuronidase/ sulfatase type H-2 from Helix pomatia | DON  DOM-1  AFM_1_  FB_1_  ZEN  α-ZEL  β-ZEL  OTA | 0.18  0.36  0.01  0.02  0.02  0.04  0.04  0.06 | 0.6  1.21  0.03  0.06  0.07  0.13  0.15  0.02 | 64-100 | 12  18  33  152  44  14  29  42 | QTrap MS/MS system (Applied Biosystems, Foster City, CA, USA), an 1100 series micro-LC system | (Gambacorta et al. 2013) |
|  |  |  |  |  |  |  |  |  | (Gambacorta et al. 2019) |
| **Dilute and Shoot approach** | - | - | DON  DON-3-Glc  DON-3-GlcAc  DOM-1 | 0.9  1.3  9.0  1.4 | 8  2.1   37.3  3.7 | 94-103 (R_E_)  56-114 (R_A_) | 56  62  72  122 | 1290 Infinity series UHPLC system (Agilent Technologies, Waldbronn, Germany) 4000 QTrap mass spectrometer | (Nagl et al. 2014) |

**Table S1a** (continued) Summary of recent sample preparation for LC-MS/MS analysis of mycotoxin biomarkers in pig urine samples.

**Table S1a** (continued) Summary of recent sample preparation for LC-MS/MS analysis of mycotoxin biomarkers in pig urine samples.

| **Sample preparation** | **Creatinine adjustment** | **Enzymatic hydrolysis (enzyme)** | **Analyte** | **LOD [ng/mL]** | **LOQ [ng/mL]** | **Recovery [%]** | **Matrix effect (SSE)  [%]** | **LC-MS** | **References** |
| --- | --- | --- | --- | --- | --- | --- | --- | --- | --- |
| **Dilute and Shoot approach** | urine diluted to the same creatinine concentration (0.2 mM) | - | DON DON-GlcAc  DON-3-S DOM-1 ZEN ZEN-14-GlcAc α-ZEL α-ZEL-14-GlcAc | - | - | 84- 115 | - | Agilent 1290 series UHPLC system coupled to a Sciex 6500 QTrap mass spectrometer | (Thanner et al. 2016) |
|  |  |  | α-ZEL  β-ZEL  ZEN  ZEN-14-Glc  ZEN-16-Glc ZEN-14-Sα-ZEL-GlcAc  β-ZEL-GlcAc ZEN-14-GlcAc | 0.11 0.16 0.15 0.07 0.18 0.02 0.73 0.42 0.30 | 0.38 0.54 0.49 0.23 0.60 0.08 2.4 1.4 1.0 | 84-113 (R_A_) | - | Agilent 1290 series UHPLC system coupled to a Sciex 6500 QTrap mass spectrometer | (Binder et al. 2017) |

| **Sample preparation** | | **Creatinine adjustment** | **Enzymatic hydrolysis (enzyme)** | **Analyte** | **LOD [ng/mL]** | **LOQ [ng/mL]** | **Recovery [%]** | **Matrix effect (SSE)  [%]** | **LC-MS** | **References** |
| --- | --- | --- | --- | --- | --- | --- | --- | --- | --- | --- |
| **Liquid-Liquid Extraction (LLE)** | ACN | - | - | T-2 HT-2 T-2 triol | 0.3 0.6  2 | 1  2 5 | 74.3 - 102.4 | 79.3- 93.8 | 1290 Infinity series UHPLC system (Agilent Technologies, Waldbronn, Germany)  4000 QTrap mass spectrometer | (Sun et al. 2014) |
|  | EtAc  (pH 2/7) | - | - | ZEN α-ZEL  α-ZAL β-ZAL  β-ZEL  ZAN  TeA AOH AME DON DOM-1 3/15-AcDON T-2 HT-2 T-2-Glc AFB_1_ AFM_1_ OTA ENA ENA_1_ ENB ENB_1_ BEA | - | 1 for all analytes  except:  DOM-1  (4 ng/ mL)   and  T-2-Glc  (2 ng/ mL) | 56.2 (T-2) - 212.8 (AOH)  (R_E_) | 90.6 84.8 85.8 85.1 85.5 90.6 52.4 11.1 73.6 79.5 5.2 72.6 90.5 100.4 107.6 72.4 79.3 106.3 81.3 80.2 83.7 82.8 89.5 | Agilent 1290 series UHPLC system coupled to a Sciex 6500 QTrap mass spectrometer | (Lauwers et al. 2019) |

**Table S1a** (continued) Summary of recent sample preparation for LC-MS/MS analysis of mycotoxin biomarkers in pig urine samples.

**Table S1a** (continued) Summary of recent sample preparation for LC-MS/MS analysis of mycotoxin biomarkers in pig urine samples.

| **Sample preparation** | | **Creatinine adjustment** | **Enzymatic hydrolysis (enzyme)** | **Analyte** | **LOD [ng/mL]** | **LOQ [ng/mL]** | **Recovery [%]** | **Matrix effect (SSE)  [%]** | **LC-MS** | **References** |
| --- | --- | --- | --- | --- | --- | --- | --- | --- | --- | --- |
| LLE | 1. step :  0.1% formic acid- ACN +  0.8 g of NaCl  2. step : upper supernatant  + 500 mg of anhydrous MgSO_4_, 50 mg of C18, 50 mg of PSA, and 50 mg of aluminia A | - | - | AFB_1_ AFB_2_ AFG_1_ AFG_2_ AFM_1_ AFM_2_ STC T-2 LYS  MET RC DAS DON 3-AcDON 15-AcDON NEO WOR VER HT-2 ZEN α-ZEL β-ZEL ZAN α-ZAL β-ZAL | - | 0.05  0.05  0.05  0.05  0.05  0.05  0.05  0.05  0.05  0.05  0.05  0.05  0.25  0.5  0.5 0.25 0.25  0.25  0.25  0.25  0.5  0.5  0.5  0.5  0.5 | 80.8 - 114.3 | signal suppression for the majority of compounds | Waters Acquity ultra-performance liquid chromatography  (UPLC) system coupled to a Micromass Quatro Micro triple quadrupole mass spectrometer (Waters, Milford, MA, USA) | (Rui Guo and Xiao Ou 2015) |

**Table S1a** (continued) Summary of recent sample preparation for LC-MS/MS analysis of mycotoxin biomarkers in pig urine samples.

| **Sample preparation** | | **Creatinine adjustment** | **Enzymatic hydrolysis (enzyme)** | **Analyte** | **LOD [ng/mL]** | **LOQ [ng/mL]** | **Recovery [%]** | **Matrix effect (SSE)  [%]** | **LC-MS** | **References** |
| --- | --- | --- | --- | --- | --- | --- | --- | --- | --- | --- |
| LLE | 1. step:  EtAc/FA (99/1, v/v) + MgSO_4_ (2 M)  2. Step: remaining aqueous phase+ ACN/FA (99/1, v/v). | - | - | DON  AFB_1_  AFM_1_ T-2 HT-2  NEO  FB_1_ OTA  OTα  ZEN α-ZEL  β-ZEL | 1 0.1  0.1 0.04  0.3 1 0.05 0.02 0.25  0.4  0.3  0.3 | 3.3 0.33  0.33  0.13 3.3 0.17  0.07  0.8  1  1.3  1 1 | 70-100 | 4 5  5 32  17 4  57  46  4  20  23  16 | Waters Acquity ultra-performance liquid chromatography  (UPLC) system coupled to a Micromass Quatro Micro triple quadrupole mass spectrometer (Waters, Milford, MA, USA) | (Song et al. 2013) |

References

Binder SB, Schwartz-Zimmermann HE, Varga E, Bichl G, Michlmayr H, Adam G, Berthiller F (2017) Metabolism of zearalenone and its major modified forms in pigs. Toxins (Basel) 9:56. https://doi.org/10.3390/toxins9020056

Brezina U, Rempe I, Kersten S, Valenta H, Humpf HU, Dänicke S (2014) Diagnosis of intoxications of piglets fed with Fusarium toxin-contaminated maize by the analysis of mycotoxin residues in serum, liquor and urine with LC-MS/MS. Arch Anim Nutr 68:425–447. https://doi.org/10.1080/1745039X.2014.973227

Gambacorta L, Olsen M, Solfrizzo M (2019) Pig urinary concentration of mycotoxins and metabolites reflects regional differences, mycotoxin intake and feed contaminations. Toxins (Basel) 11:378. https://doi.org/10.3390/toxins11070378

Gambacorta S, Solfrizzo H, Visconti A, Powers S, Cossalter AM, Pinton P, Oswald IP (2013) Validation study on urinary biomarkers of exposure for aflatoxin B _1_ , ochratoxin A, fumonisin B _1_ , deoxynivalenol and zearalenone in piglets. World Mycotoxin J 6:299–308. https://doi.org/10.3920/WMJ2013.1549

Jodlbauer J, Zöllner P, Lindner W (2000) Determination of zeranol, taleranol, zearalenone, α- and β-zearalenol in urine and tissue by high-performance liquid chromatography-tandem mass spectrometry. Chromatographia 51:681–687. https://doi.org/10.1007/BF02505405

Lauwers M, De Baere S, Letor B, Rychlik M, Croubels S, Devreese M (2019) Multi LC-MS/MS and LC-HRMS methods for determination of 24 mycotoxins including major phase I and II biomarker metabolites in biological matrices from pigs and broiler chickens. Toxins (Basel) 11:171. https://doi.org/10.3390/toxins11030171

Nagl V, Woechtl B, Schwartz-Zimmermann HE, Hennig-Pauka I, Moll WD, Adam G, Berthiller F (2014) Metabolism of the masked mycotoxin deoxynivalenol-3-glucoside in pigs. Toxicol Lett 229:190–197. https://doi.org/10.1016/j.toxlet.2014.06.032

Rui Guo W, Xiao Ou S (2015) Simultaneous Detection Method for Mycotoxins and their Metabolites in Animal Urine by Using Impurity Adsorption Purification followed by Liquid Chromatography-Tandem Mass Detection. J Chromatogr Sep Tech 6:308. https://doi.org/10.4172/2157-7064.1000308

Song S, Ediage EN, Wu A, De Saeger S (2013) Development and application of salting-out assisted liquid/liquid extraction for multi-mycotoxin biomarkers analysis in pig urine with high performance liquid chromatography/tandem mass spectrometry. J Chromatogr A 1292:111–120. https://doi.org/10.1016/j.chroma.2012.10.071

Sun Y, Zhang G, Zhao H, Zheng J, Hu F, Fang B (2014) Liquid chromatography-tandem mass spectrometry method for toxicokinetics, tissue distribution, and excretion studies of T-2 toxin and its major metabolites in pigs. J Chromatogr B Anal Technol Biomed Life Sci 958:75–82. https://doi.org/10.1016/j.jchromb.2014.03.010

Thanner S, Czeglédi L, Schwartz-Zimmermann HE, Berthiller F, Gutzwiller A (2016) Urinary deoxynivalenol (DON) and zearalenone (ZEA) as biomarkers of DON and ZEA exposure of pigs. Mycotoxin Res 32:69–75. https://doi.org/10.1007/s12550-016-0241-2
